# Impact of loneliness on health in healthy populations: A meta‐analysis

**DOI:** 10.1111/bjhp.70040

**Published:** 2025-12-07

**Authors:** Yixuan Zheng, Pamela Qualter, Cecilia Rollano, Xinyang Hu, Arianna Martin, Maria Nukui, Wahida Walibhai, Marlies Maes

**Affiliations:** ^1^ Manchester Institute of Education University of Manchester Manchester UK; ^2^ College of Teacher Education, Taishan University Tai’an China; ^3^ Faculty of Education National University of Distance Education Madrid Spain; ^4^ Interdisciplinary Social Science: Youth Studies Utrecht University Utrecht The Netherlands

**Keywords:** health, health service use, healthy populations, hearing acuity, loneliness, sleep, visual acuity

## Abstract

**Objectives:**

Previous meta‐analyses have shown that loneliness is associated with poor health (*r* = −.35) and sleep challenges (*r* = .29). However, that work is confounded by the inclusion of unhealthy populations, with chronic conditions, such as HIV, Alzheimer's disease, diabetes, and severe mental illness. This pre‐registered meta‐analysis (PROSPERO CRD42019119135) examined whether loneliness is linked to poorer health in healthy populations.

**Design:**

Meta‐analysis.

**Methods:**

PsycINFO, ERIC, PubMed, and Web of Science were searched for articles before January 2024 to examine the association between loneliness and health outcomes (categorized as general health, physical health [functional disability], sleep function, sensory acuity, and health service use) for healthy samples.

**Results:**

A total of 167 articles (303,643 participants; 334 effect sizes; 158 independent samples) from 36 countries were included in the meta‐analysis. Loneliness is associated with worse health for healthy populations (*r* = −.22), with the largest effect (*r* = −.23) for general health. Loneliness was not related to health service use for healthy populations. Heterogeneity was substantial, with only 7.2% of the total variance attributable to sampling error, indicating that over 92% of the variability in effect sizes reflected true differences across studies.

**Conclusions:**

Higher loneliness was associated with poorer health for healthy people. There are limited studies using (1) objective measurements of health outcomes in relation to loneliness, and (2) studies exploring the association among young people, highlighting a need for more work in those areas.

**Statement of Contribution**

**What is already known on this subject?**

- Loneliness has been widely associated with poor health outcomes, and this relationship has been recognized as a public health issue globally. However, prior meta‐analyses included individuals with chronic illnesses, limiting the generalizability of findings to the general healthy population. Those analyses have emphasized the negative impact of loneliness on health, but often confounded the relationship with pre‐existing health conditions.

**What does this study add?**

- This study is the first comprehensive meta‐analysis to focus solely on healthy populations and demonstrated that loneliness significantly correlates with poorer health outcomes, including general health, physical limitations, and sleep function. It highlights that those effects are robust even in non‐clinical samples, underscoring the broader public health implications of loneliness.
- The study showed that self‐reported health outcomes show stronger associations with loneliness than objective measures of health. This nuanced understanding highlights the complexity of the loneliness–health relationship.

## INTRODUCTION

Loneliness is a common experience across the lifespan (Qualter et al., 2015) and across the world (Barreto et al., 2021). It is a subjective experience that includes a cognitive component (the perception that one's social relationships are not what one wants) and an affective one (the presence of sadness, anger, or another negative emotion) (Badcock et al., 2022). Loneliness is measured using self‐report questionnaires, which often include multiple questions that indirectly ask about the frequency of loneliness (Maes et al., 2022). Loneliness is different from social isolation. Social isolation is conceptualized as the amount of time one spends alone and the frequency with which one engages in social interaction with others in one's network (Lim et al., 2020) being measured by asking people how often they see or hear from friends, family, and neighbours in any given month.

Meta‐analyses have shown negative associations between loneliness and health outcomes (Park et al., 2020) and positive associations between loneliness and poor sleep (Griffin et al., 2020; Park et al., 2020). Specifically, the effect sizes (*r*) were general health = −.35, physical health = −.30, sleep challenges = .29, impaired sleep quality = .26, insomnia symptoms = .28. Such associations are evident across the lifespan, with general health, particularly self‐reported general health (e.g., stomach aches and headaches), and poor, disturbed sleep, being problems for those reporting loneliness across ontogeny. Because of that association, loneliness has been declared a public health issue in many countries (Holt‐Lunstad et al., 2017), with several countries developing government policies that seek to address loneliness (Goldman et al., 2024). The recent World Health Organization Commission on Social Connection reinforces that global prioritization, identifying social disconnection – including loneliness – as a serious public health issue and calling for urgent, coordinated action across policy, research, and community domains (World Health Organization, 2025).

Despite the novelty of previous meta‐analyses and their important contribution, the presence of individuals with chronic health conditions in samples makes it difficult to ascertain whether loneliness was an issue for just those with chronic conditions, or also an issue for healthy, yet lonely, people. Essentially, the inclusion of patients with chronic conditions (e.g., HIV, cancer, Alzheimer's disease, severe mental illness, etc.) makes it difficult to be certain that loneliness is a health concern for the general population.

The inclusion of unhealthy individuals in earlier meta‐analyses may introduce potential biases because those samples are less representative of the general population. Specifically, the presence of potentially confounding factors – such as pre‐existing health conditions or associated risk factors – complicates the interpretation of the relationship between loneliness and health outcomes. These factors may be associated with both loneliness and poorer health, making it difficult to isolate the unique contribution of loneliness without more rigorous adjustment or stratification. Having unhealthy individuals in samples may, thus, underestimate the true effects of loneliness on health across the entire population, potentially leading to interventions that are successful with only a specific subset of individuals. Consequently, findings from studies with predominantly unhealthy samples may lack generalizability to healthier populations, hindering a comprehensive understanding of the impact of loneliness on health across diverse societal groups. The extrapolation of findings from potentially biased samples to the broader population poses challenges and may result in misguided policies or interventions that inadequately address the needs of healthier individuals. Addressing those potential biases underscores the importance of conducting research into loneliness and health using healthy samples to ensure the robustness and applicability of findings in informing effective interventions targeting loneliness‐related health issues for the general population.

Loneliness warrants more attention as an indicator of general population health. In the current meta‐analysis, we examine only those studies that included a non‐clinical, general population sample.

## METHODS

This systematic review was conducted and reported in accordance with the PRISMA 2020 guidelines. The protocol for this review was registered in Prospero (CRD42019119135).

### Search for studies

First, we used the MASLO database (Maes, Qualter, et al., 2019) to find papers up to 2016 that explored loneliness and health. MASLO used a broad literature search of databases (PsycINFO, ERIC, PubMed, and Web of Science) using key terms that reflect the names of the eight main loneliness questionnaires, with the aim of retrieving all studies in which those questionnaires had been used. A full list of key terms used to create the MASLO dataset can be found at the Open Science Framework (https://osf.io/8ab56/files/nv9hm). We began by searching the MASLO dataset for studies examining the relationship between loneliness and health, using the keywords ‘*loneliness*’ and ‘*health*’. From the papers identified in that initial search, we derived additional search terms to refine and update our search, with a specific focus on loneliness and health in healthy populations. We applied this updated search strategy across four databases: PsycINFO, ERIC, PubMed, and Web of Science. The full list of key search terms used in those databases can be found here: https://osf.io/r7km6. The final update of our search took place in January 2024; when we combined the papers from the MASLO dataset with those we had identified subsequently, we had identified 4079 for screening.

### Inclusion and exclusion criteria

The following selection criteria were used: The study had to be published in English and in a peer‐reviewed journal. Empirical, quantitative studies were included, but systematic reviews, meta‐analyses, and qualitative studies were not. Regarding the study sample/population, no criteria were set for age, ethnicity, or other demographic characteristics, except that participants had to be from healthy populations; studies involving those from clinical or otherwise unhealthy samples were excluded. There were some instances where people were no longer unhealthy (e.g., samples of people who had recovered from cancer and no longer needed treatment); for our purposes, those samples were counted as currently healthy, and the studies included in the meta‐analysis.

Regarding the key variables – loneliness and health – we followed strict criteria for inclusion. First, studies had to focus on loneliness, with those focused only on social exclusion, social isolation, and other aspects of social connection being excluded; the studies also had to collect data on loneliness using one of the eight main loneliness measures categorized in MASLO. For health, included studies had to measure health as fitness, disease, or proper functioning of bodily systems; papers that described health in terms of mental health and well‐being were excluded. The articles that included a measure of Quality of Life as a health outcome, but did not examine the separate subscales, so that mental health and/or well‐being were considered separately, were excluded.

### Study screening

**FIGURE 1:**
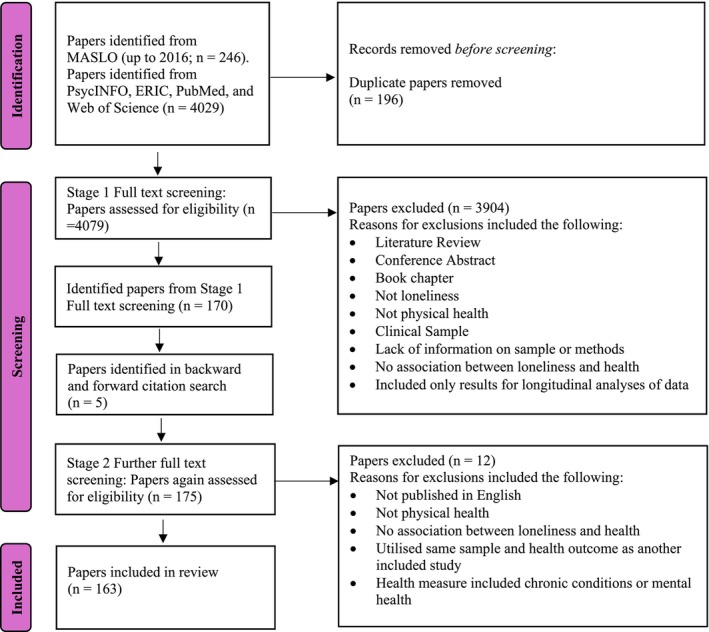
PRISMA diagram showing the identification, screening, eligibility assessment, and inclusion of studies examining loneliness and health in healthy populations.

The search of the MASLO dataset and the updated searches resulted in 4275 records. There were 196 duplicate records, and those were removed at this initial stage before screening. In Stage 1 of screening, we screened the full texts of the 4079 articles from our searches, excluding 3908 for not meeting the eligibility criteria. To ensure consistency, teams of reviewers independently screened a random subset of 20 articles, achieving 90% agreement. Disagreements and uncertainties were discussed until full consensus (100% agreement) was reached. The remaining records were then divided equally among the reviewers for independent screening. In Stage 2 of screening, we (YZ and WW) screened the full texts of the 170 articles we had identified from Stage 1 screening and the 5 articles we had identified from our forward and backward citation searches. The PRISMA diagram (Figure 1) shows that data from 163 articles were eligible to be extracted. All screening and reference management were conducted using EndNote20.

### Data extraction and coding

Data from the final papers were extracted. The code books for the current study are available at https://osf.io/8ab56/overview. Data included sample information (the age of participants, the percentage of females in the sample, and socio‐economic representation), loneliness data (loneliness measure used, including whether it was the full version or a shorter version of the measure), health outcome explored, and whether that was objectively or subjectively measured. We classified health outcomes as follows: general health (e.g., overall health, BMI, blood pressure), physical health (e.g., physical limitations, mobility), use of services (e.g., GP visits, use of ambulance), sleep function (e.g., sleep disturbance, sleep quality) and sensory acuity (vision accuracy, hearing difficulties). Health outcomes were coded as *subjective* when they relied on participants' self‐assessments or self‐reports (e.g., self‐rated health, self‐reported BMI), and as *objective* when derived from externally verified or clinician‐measured data (e.g., medical records, physiological measures). Additional study characteristics included the year of publication and the country where the study was conducted. We also noted whether the effect sizes were obtained from the same sample of participants – that could happen within the same paper, but also in cases where the same data source (e.g., population sample) was used in several studies across several articles.

In the manuscript, the reader will see reference to ‘overall health’ and ‘global health’. Please note that ‘overall health’ refers to an individual's physical well‐being (forms part of the general health outcome measure as noted above), whereas ‘global health’ reflects a composite measure derived from the multiple health outcomes, and which is measured in the meta‐analyses.

### The final set of studies

The final dataset consisted of 163 articles published between 1982 and January 2024 that included data related to the concurrent relationship between loneliness and health; the articles reported 340 effect sizes from 175 independent samples. Study characteristics are detailed in Table S1. Studies came from 36 countries, with most effect sizes from the United States (*k* = 124). Sample sizes ranged from 23–114,428 participants, with a total of 18,808 children and adolescents, 179,533 adults and 112,624 older adults; 61.4% of participants were females. Loneliness was always measured using self‐reports; health was measured by self‐assessment or self‐reports for 266 of the effect sizes, and objectively (derived from externally verified or clinician‐measured data) in 74.

### Data analysis plan

In this meta‐analysis, Pearson's *r*, the correlation coefficient between loneliness and health outcome, was used as the primary effect size metric because it was most commonly reported in the studies. Effect sizes were interpreted according to contemporary benchmarks (Funder & Ozer, 2019), where an *r* value of .05 was considered very small, .10 as small, .20 as medium, .30 as large, and .40 or greater as very large. For studies that did not report Pearson's *r* directly, we converted alternative statistics to *r* values. For example, odds ratios were first transformed into *d* values and then into *r*. To correct for potential skewness in the distribution of Pearson's *r*, we applied Fisher's *Z* transformation and then converted those *Z* values back to *r* for easier interpretation. Additionally, to maintain consistency, we adjusted the direction of correlations from studies with negative health scales so that all effect sizes consistently reflected the relationship between loneliness and better health.

Given that multiple correlation coefficients from the same sample can create dependencies between effect sizes – and that multiple samples drawn from the same dataset (e.g., different waves or age groups) often share similar sampling procedures or use the same questionnaire – these factors may undermine the assumption of independence in traditional meta‐analyses (Lipsey & Wilson, 2001). To retain important data and avoid a reduction in statistical power, we employed a multilevel modelling approach. Specifically, we used a four‐level structure in our meta‐analytic model to account for variance at four levels: sampling variance at Level 1, variance between effect sizes from the same sample at Level 2, variance between the samples with the same data source at Level 3, and variance between data sources at Level 4. This four‐level random effects model was implemented using the metafor package in R (Viechtbauer, 2010), with parameters estimated through restricted maximum‐likelihood (REML). We selected REML because it is considered an unbiased estimator and empirical evidence has demonstrated its superior performance compared with other estimators (Hönekopp & Linden, 2022; Langan et al., 2019; Viechtbauer & Cheung, 2010). Heterogeneity across levels was examined using likelihood ratio tests (LRTs) for each random‐effect variance component, and by reporting the total residual heterogeneity statistic (*Q*), its significance (*p*), and the proportion of total variance attributable to true heterogeneity (*I* ^2^).

To ensure the robustness of our results, we also assessed the influence of outliers using the influence function of the metafor package to compute leave‐one‐out diagnostics, reporting the overall effect sizes and conducting moderator analyses with and without the inclusion of potential outliers. The moderators were tested separately in each model and included publication year, continent, gender, loneliness scale, type of health outcome measurement (objective or subjective), age category, and socio‐economic status (SES). There are only a small number of effect sizes for the use of services, sleep function (e.g., sleep disturbance, sleep quality), and sensory acuity (vision accuracy, hearing difficulties), so while we report findings of moderator analyses in the tables, we are cautious in making conclusions. For the global health, general health, and physical health domains, which included sufficient effect sizes, only categories represented by at least six effect sizes were included in the moderator analyses to ensure stable estimation (Maes, Nelemans, et al., 2019).

In the meta‐analysis, *global health* refers to a composite indicator capturing health status as reported across the included studies. This construct does not focus on a single domain (e.g., physical health, use of services, sleep function, or sensory acuity), but rather represents an aggregate or summary index of all the health outcomes. Global Health essentially refers to the pooled analysis of all health outcomes combined in the initial meta‐analysis, prior to examining specific health domains separately (e.g., general health, physical health, sleep function).

To examine the presence of publication bias, we created funnel plots using Fisher's *Z* transformations, which we would expect to be shaped as a symmetric inverted funnel if publication bias is absent (Torgerson, 2006). We conducted Egger's regression test to statistically test the asymmetry of the funnel plots (Egger et al., 1997).

## RESULTS

Table 1 shows the average effect sizes for the different health domains, and the average effect size of the association between loneliness and *global health*. Analysis of the association between loneliness and global health based on the random effects model yielded an estimated mean Fisher's *Z* of −.22 (SE = .015, *p* < .0001), which back‐transformed to a Pearson correlation corresponding to a mean estimated effect size of *r* = −.21, 95% CI = [−.24, −.19]. That result indicates a medium effect size, whereby higher levels of loneliness are significantly associated with poorer overall health outcomes among healthy samples.

**TABLE 1:** Effect sizes for the association between loneliness and health domains.

Health Domain	*k*	Fisher's *Z* (95% CI)	*SE*	*p*	*r* (95% CI)	*I* ^2^	*Q* (*df*)
Global health	340	−**.2181** (−.2468, −.1895)	.0146	<.0001	−.2148 (−.2419, −.1873)	91.91	16682.0640 (339)
General health	184	−**.2340** (−.2724, −.1957)	.0195	<.0001	−.2299 (−.2659, −.1932)	92.70	6428.9619 (183)
Physical health	75	−**.2001** (−.2561, −.1441)	.0281	<.0001	−.1975 (**−**.2507, −.1431)	94.85	6655.4604 (74)
Use of services	11	−.0938 (−.2266, .0390)	.0596	.1467	−.0935 (−.2228, .0390)	94.81	2010.4765 (10)
Sleep function	50	−**.1954** (−.2473, ‐.1436)	.0258	<.0001	−.1930 (−.2424, −.1426)	90.42	659.2038 (49)
Sensory acuity	20	−**.1187** (−.1630, −.0743)	.0212	<.0001	−.1181 (−.1616, −.0742)	74.73	160.1823 (19)

*Note:* *p* < .05 is significant, k = number of effect sizes, CI = confidence interval, *SE* = standard error, *I* ^*2*^ = percentage of variation due to variability of true effects, *Q* = test for residual heterogeneity (*df* = k − 1), all *Q*‐tests were significant at *p* < .0001. Figures in bold indicate statistical significance.

Specifically, a significant medium effect was found for the relationship between loneliness and general health (Fisher's *Z* = −.23 (SE = .020, *p* < .0001); *r* = −.23, 95% CI = [−.27, −.19]), and significant small‐to‐medium effects were found for the relationships between loneliness and physical health (Fisher's *Z* = −.20 (SE = .028, *p* < .0001); *r* = −.20, 95% CI = [−.25, −.14]), and sleep function (Fisher's *Z* = −.20 (SE = .026, *p* < .0001); *r* = −.19, 95% CI = [−.24, −.14]). The association between loneliness and sensory acuity was small (Fisher's *Z* = −.12 (SE = .021, *p* < .0001); *r* = −.12, 95% CI = [−.16, −.07]). The only association that did not reach significance was between loneliness and use of services (Fisher's *Z* = −.09 (SE = .06, *p* = .15); *r* = −.09, 95% CI = [−.22, −.04]).

When examining heterogeneity across hierarchical levels for the overall model, the median sampling variance was 0.0031, accounting for 8.09% of the total variance. The Level 2 variance was 0.0188, LRT *χ* ^*2*^ (1) = 39.20, *p* < .0001, accounting for 48.55% of the total variance, indicating substantial within‐sample variation. The Level 3 variance was 0.0000, LRT *χ* ^*2*^ (1) = 0.00, *p* = 1.00, accounting for 0.00% of the variance, suggesting no meaningful heterogeneity at this level. This likely reflects high similarity among samples drawn from the same dataset, which may share common measurement instruments, sampling procedures, and study contexts, leading to small between‐sample differences. The Level 4 variance was 0.0168, LRT *χ* ^*2*^ (1) = 3015.59, *p* < .0001, accounting for 43.35% of the total variance, indicating strong between‐dataset heterogeneity. These patterns remained consistent after outlier removal (see Supplementary Material S2 for details on the full model, outlier exclusion, and domain‐specific analyses).

In terms of total heterogeneity, only 8.09% of the total variance was attributable to sampling error, indicating that *I* ^*2*^ = 91.91% of the heterogeneity was due to true variation in effect sizes. The overall test of residual heterogeneity was also significant, *Q* (*df* = 339) = 16682.06, *p* < .0001, confirming the presence of considerable true between‐effect variability. In multilevel meta‐analysis, heterogeneity is generally considered substantial if Level 1 sampling variance accounts for less than 75% of the total variance (Assink & Wibbelink, 2016; Hunter & Schmidt, 2004). This pattern was also observed when examining different health domains (see Table 1).

Given the observed heterogeneity, we conducted moderator analyses based on sample, measure, and contextual characteristics to explore potential sources of variation in the strength of associations between loneliness and different health domains. Although Level 3 variance was negligible in the overall model, the four‐level specification was retained for two reasons: (1) to maintain conceptual consistency with the hierarchical data structure, which included multiple samples nested within datasets; and (2) to ensure comparability with subgroup and moderator analyses, some of which (e.g., the general health domain) showed non‐trivial Level 3 variance (14.42% of the total variance; see Supplementary Material S2). We therefore retained the four‐level model for consistency but interpreted heterogeneity primarily at the within‐sample (Level 2) and between‐dataset (Level 4) levels, where the most meaningful variation occurred.

### Moderation analyses

Moderators were tested using linear regression models and most of the moderators did not reach significance (see Table S2 for full information). Only one moderator significantly affected the strength of the relationship between loneliness and global health and that was the type of health outcome measurement (*F*(1, 338) = 17.01, *p* < .0001): effect sizes were greater when health outcomes were reported subjectively via self‐report (*r* = −.23, *p* < .001, 95% CI = [−.26, −.20]) compared with when they were reported by an objective measure (e.g., blood pressure, gut microbiome; *r* = −.12, *p* < .001, 95% CI = [−.17, −.07]).

When exploring the different health domains, the type of health outcome measurement was a significant moderator of the relationship between loneliness and general health (*F*(1, 182) = 17.12, *p* < .0001) following the same pattern of medium effect sizes for subjectively measured health outcomes (*r* = −.26, *p* < .001, 95% CI = [−.30, −.22]), but this time only very small effect sizes for objectively measured health outcomes (*r* = −.09, *p* < .05, 95% CI = [−.17, −.01]). Furthermore, continent was a significant moderator of the relationship between loneliness and sensory acuity (*F*(2, 17) = 6.2044, *p* < .05), with only studies from Europe (*r* = −.12, *p* < .01, 95% CI = [−.19, −.06]) and North America (*r* = −.13, *p* < .001, 95% CI = [−.17, −.09]) yielding significant effect sizes, but not studies from Asia. However, due to the small number of effect sizes measuring sensory acuity, caution should be taken from drawing conclusions from this result. Analyses for relationships between loneliness and physical health, sleep function and use of services yielded no significant moderators.

### Influential cases and outliers

Analysis using the influence function in the metafor package detected six influential cases (Effect IDs 104, 186, 188, 189, 196 and 267). Four of the effect sizes were in the general health domain and two measured physical health. After deleting those outliers and re‐running the analysis, the overall effect size of the relationship between loneliness and *global health* decreased in magnitude (Fisher's *Z* = −.20 (SE = .011, *p* < .0001); *r* = −.20, 95% CI = [−.22, −.18]). Specifically, the effect sizes of the relationships between loneliness and general and physical health reduced (see Table 2).

**TABLE 2:** Effect Sizes for the Association Between Loneliness and Health Domains After Deleting Outliers.

Health domain	k	Fisher's *Z* (95% CI)	*SE*	*p*	*r* (95% CI)	*I* ^*2*^	*Q* (*df*)
Global health	334	−**.2021** (−.2232, −.1809)	.0107	<.0001	−.1994 (−.2195, −.1790)	84.70	7901.0269 (333)
General health	180	−.**2130** (−.2408, −.1852)	.0141	<.0001	−.2098 (−.2362, −.1831)	85.48	2773.3243 (179)
Physical health	73	−**.1749** (−.2097, −.1401)	.0175	<.0001	−.1731 (−.2067, −.1391)	84.90	1280.5354 (72)
Use of services	11	−.0938 (−.2266, .0390)	.0596	.1467	−.0935 (−.2228, .0390)	94.81	2010.4765 (10)
Sleep function	50	−**.1954** (−.2473, −.1436)	.0258	<.0001	−.1930 (−.2424, −.1426)	90.42	659.2038 (49)
Sensory acuity	20	−**.1187** (−.1630, −.0743)	.0212	<.0001	−.1181 (−.1616, −.0742)	74.73	160.1823 (19)

*Note:* *p* < .05 is significant, k = number of effect sizes, CI = confidence interval, *SE* = standard error, *I* ^*2*^ = percentage of variation due to variability of true effects, *Q* = test for residual heterogeneity (*df* = *k* − 1), all *Q*‐tests were significant at *p* < .0001. Figures in bold indicate statistical significance.

Following the deletion of the influential cases, the same moderator of type of health outcome measurement continued to be important moderator for global health (*F*(1, 332) = 24.60, *p* < .0001) and general health (*F*(1, 179) = 25.75, *p* < .0001), following the same pattern of medium effect sizes for subjectively measured health outcomes, but only small or very small effect sizes for objectively measured health outcomes. (see Table S2).

### Publication bias

All funnel plots of effect sizes by their standard errors showed a roughly symmetrical shape, suggesting that publication bias was not present (see Figures S1 and S2). Egger's regression test further confirmed a lack of strong asymmetry in the funnel plots when outliers were included (*z* = −0.12, *p* = .907) and deleted (*z* = −1.33, *p* = .183).

## DISCUSSION

We found a significant relationship between loneliness and poorer health outcomes in otherwise healthy individuals. The findings contribute to a growing body of evidence suggesting that social relationships play a crucial role in overall health. The implications are profound, highlighting the need to address loneliness as a public health priority. While previous research has explored the link between loneliness and health, with governments beginning to recognize loneliness as a public health concern, our meta‐analysis is the first to synthesize the research with healthy individuals, demonstrating the significant impact of loneliness on health for the general population.

### Main findings

First, our findings show that the magnitude of the association between loneliness and health outcomes is reduced when analyses are restricted to healthy populations. Previous meta‐analytic studies, which included participants with chronic health conditions, reported stronger associations between loneliness and both general health (*r* = −.35) and sleep (*r* = −.29). In contrast, the present analysis, which excluded such populations, identified a more modest, but still significant association between loneliness and overall health (*r* = −.22), with the strongest effect observed for general health outcomes (*r* = −.23). These attenuated effect sizes suggest that prior estimates may have been inflated by the inclusion of individuals with existing health vulnerabilities that both exacerbate and are exacerbated by loneliness. By focusing exclusively on healthy individuals, this meta‐analysis offers a more conservative and precise estimate of the relationship between loneliness and health, underscoring the need to consider baseline health status in both research and intervention design.

Our analyses revealed consistent evidence across multiple studies that loneliness is associated with negative health outcomes in individuals who are considered healthy. This relationship was observed across a range of health measures, including physical health indicators such as physical limitations in daily activities, and general health complaints. Importantly, we found that the magnitude of those effects did not decrease when moderators were included in the model; that included SES, a well‐established determinant of health that influences access to healthcare, nutrition, living conditions, and stress levels. These findings suggest that the health issues associated with loneliness are not fully explained by socio‐economic factors.

We found that loneliness was associated with poorer health in healthy samples, but that the magnitude of the effect was higher when subjective reports of general health were used to measure compared with when objective measures like blood pressure were taken. The reason loneliness may show stronger associations with subjective reports of general health than with objective indicators like blood pressure is that both loneliness and self‐rated health are subjective experiences shaped by individual perceptions and emotions. People who feel lonely may be more likely to interpret bodily sensations negatively or report feeling generally unwell, even in the absence of clinical symptoms; conversely, individuals experiencing poor health may also report more loneliness due to reduced opportunities or capacity for social connection. This shared reliance on self‐perception can inflate the correlation between loneliness and subjective health measures. In contrast, objective health indicators such as blood pressure or biomarkers are less influenced by mood, cognition, or social context, and may not capture the more diffuse or early‐stage physiological effects of loneliness, partly because a comprehensive bio‐behavioural theory explaining those mechanisms is still lacking. Thus, while loneliness may contribute to physiological dysregulation over time, its immediate impact looks to be more detectable through self‐reported health.

We also found a moderating effect of continent on sensory acuity, suggesting potential cultural or contextual differences in how loneliness relates to sensory functioning. It is possible that, in Western contexts, sensory decline may more strongly disrupt social interaction and emotional well‐being; in some Asian contexts, family and community structures may buffer against those effects, or loneliness may be conceptualized and expressed differently. However, this interpretation should be treated with caution given the limited number of studies assessing sensory acuity and the uneven regional distribution of data: the apparent continental differences may reflect sampling variation, measurement non‐equivalence, or other contextual factors rather than genuine cultural distinctions. Further research using culturally validated measures is needed to clarify whether the regional patterns represent true differences in the loneliness–sensory acuity association.

### What is already known on this topic

Previous research has established a link between loneliness and poor health. Such work is often highlighted by governments (Goldman et al., 2024) keen to address the negative impact of loneliness on health often with a specific focus on reducing health care costs. While that work has increased public awareness of the issue, it is limited because it includes unhealthy individuals and does not control for confounding variables that impact loneliness and poor health. Our analysis shows that even in healthy populations, loneliness is linked to poorer health, although we did not find that loneliness was associated with health service use. Thus, our findings show the generalizability of findings linking loneliness and poorer health to the general population.

### What this study adds

Our findings that loneliness is associated with poorer health among healthy populations has significant public health implications. Given the pervasive nature of loneliness, there is a critical need for interventions aimed at reducing loneliness. Such interventions could include community‐based programmes that promote social engagement, mental health services that address feelings of loneliness, and policies that foster inclusive and supportive environments. Healthcare providers should also be aware of the impact of loneliness on health and consider it when assessing patient well‐being. Routine screening for loneliness in healthcare settings could facilitate timely intervention.

Understanding the mechanisms linking loneliness and health is also critical. We found that objective measures of health are less significantly associated with loneliness than subjective reports of health, suggesting that subjective reports offer important insights into how loneliness relates to an individual's overall sense of well‐being. It is possible that the stronger associations observed between loneliness and subjectively reported health outcomes reflect shared variance with broader affective tendencies, such as negative affectivity (Watson & Pennebaker, 1989). This underscores the importance of considering both subjective and objective indicators when examining how loneliness relates to an individual's overall sense of well‐being. That finding, then, highlights key factors that should be considered in public health programmes for loneliness: (1) loneliness may lead individuals to rate their health more negatively due to their emotional state, even if objective measures do not indicate poor health; (2) subjective health reports are influenced by an individual's mood, mindset, and personal experiences, with loneliness potentially amplifying perceived health problems through stress, anxiety, or depression; and (3) objective health measures might not capture certain dimensions of well‐being that are affected by loneliness, such as social functioning/support, emotional well‐being, or energy levels.

Furthermore, given that individuals with low well‐being are more likely to engage in unhealthy behaviour such as poor diet, physical inactivity, smoking, and excessive alcohol consumption (Grant et al., 2009), it is crucial to explore how loneliness may contribute to those behaviours. Understanding such mechanisms would greatly enhance our ability to develop effective interventions that address both loneliness and its associated health risks.

### Limitations

Our analyses provide robust evidence of the relationship between loneliness and health, but there are some limitations. First, the studies included in this analysis varied in their definitions and measurements of loneliness, which introduced heterogeneity into the findings. Future research should aim to standardize the measurement of loneliness and explore the mechanisms through which loneliness affects health. Longitudinal studies would also be particularly valuable in establishing causal relationships and identifying potential intervention points. We also note that there were limited studies (1) using objective measurements of health outcomes in relation to loneliness for healthy individuals, and (2) with young people, highlighting a need for more work in those areas.

## CONCLUSION

In conclusion, this meta‐analysis underscores the significant relationship between loneliness and poor health outcomes in healthy individuals. Our findings highlight the importance of addressing loneliness as a public health issue and suggest that interventions aimed at reducing loneliness could have substantial benefits for individual and population health and well‐being. As our understanding of the health correlates of loneliness continues to grow, it is imperative that we develop and implement effective strategies to combat it. Addressing loneliness improves well‐being and reduces health problems associated with it.

## AUTHOR CONTRIBUTIONS

**Yixuan Zheng:** Methodology; data curation; investigation; validation; formal analysis; visualization; writing – review and editing; project administration; supervision. **Pamela Qualter:** Conceptualization; methodology; data curation; investigation; validation; supervision; project administration; writing – original draft; writing – review and editing; resources; visualization. **Cecilia Rollano:** Data curation; investigation; formal analysis; validation; writing – review and editing; supervision. **Xinyang Hu:** Data curation; investigation; validation; writing – review and editing; writing – original draft. **Arianna Martin:** Data curation; investigation; validation; writing – review and editing; writing – original draft. **Maria Nukui:** Data curation; investigation; validation; writing – review and editing; writing – original draft. **Wahida Walibhai:** Data curation; investigation; validation; writing – original draft; writing – review and editing; visualization. **Marlies Maes:** Conceptualization; methodology; data curation; investigation; validation; writing – original draft; writing – review and editing; resources; supervision.

## FUNDING INFORMATION

This research was not funded.

## CONFLICT OF INTEREST STATEMENT

The authors report no conflict of interest.

## Supporting information


Data S1:

Data S2:

Data S3:

## Data Availability

Data are available at https://osf.io/8ab56/.
