# Supplementary material for: Impact of loneliness on health in healthy populations: A meta‐analysis
Source: Br J Health Psychol. 2025 Dec 7;31(1):e70040. doi: 10.1111/bjhp.70040 (PMC12683082; doi:10.1111/bjhp.70040)
Supplement: Supplementary file 1 — Data S1: [file BJHP-31-0-s001.docx]

**Supplementary Material S1**

**The Association Between Loneliness and Health for Healthy Populations**

**Table S1** Summary of Studies Included in Meta-Analyses

| Effect Size ID | Authors (Year) | Sample Characteristics | | | | | | Measure Characteristics | | | | Effect Size |
| --- | --- | --- | --- | --- | --- | --- | --- | --- | --- | --- | --- | --- |
|  |  | Country | Continent | Number of Participants | Gender | Age Category | Socioeconomic Status | Loneliness Scale | Health Domain | Measure of Health Outcome | Type of Health Outcome Measurement |  |
| 1 | Adams et al. (2004)^1^ | United States | North America | 234 | 0.74 | Older Adults | Mixed | UCLA | General health | Self-rated health | Subjective | -.2310 |
| 2 | Auslander et al. (2003)^2^ | Israel | Asia | 181 | 0.73 | Older Adults | Mixed | UCLA | Use of services | On-call physician | Subjective | -.2250 |
| 3 |  |  |  |  |  |  |  | UCLA | Use of services | Distress button | Subjective | -.3020 |
| 4 |  |  |  |  |  |  |  | UCLA | Use of services | Use of ambulance | Subjective | -.2280 |
| 5 |  |  |  |  |  |  |  | UCLA | General health | Self-rated health | Subjective | -.2970 |
| 6 |  |  |  |  |  |  |  | UCLA | Physical health | Functional limitations- need for assistance with daily activities | Subjective | -.3470 |
| 7 | Baum (1982)^3^ | United States | North America | 75 | 0.51 | Older Adults | - | UCLA | General health | Frequency of illness, health problems (including all aspects of health except mental health). | Subjective | -.3600 |
| 8 | Bookwala (2011)^4^ | United States | North America | 738 | 0.40 | Older Adults | Middle/High | UCLA | Physical health | Self-rated physical limitations | Subjective | -.1100 |
| 9 |  |  |  |  |  |  |  | UCLA | Sensory | Vision acuity test | Objective | -.0100 |
| 10 |  |  |  |  |  |  |  | UCLA | Sensory | Self-rated vision | Subjective | -.1700 |
| 11 | Bookwala & Lawson (2011)^5^ | United States | North America | 1170 | 0.51 | Older Adults | Middle/High | UCLA | Sensory | Visual acuity test | Objective | -.0800 |
| 12 |  |  |  |  |  |  |  | UCLA | Sensory | Self-rated vision | Subjective | -.2000 |
| 13 |  |  |  |  |  |  |  | UCLA | Physical health | Self-rated physical limitations | Subjective | -.1500 |
| 14 | Chalise et al. (2007)^6^ | Nepal | Asia | 195 | 0.48 | Older Adults | Low | UCLA | General health | Self-reported overall health | Subjective | -.2590 |
| 15 |  |  |  |  |  |  |  | UCLA | Physical health | Physical limitations- Intrumental Activities of Daily Living (IADL) | Subjective | -.1430 |
| 16 | Downs et al. (1988)^7^ | United States | North America | 100 | 0.79 | Older Adults | Middle/High | UCLA | General health | Overall health | Subjective | -.1660 |
| 17 | Durak & Senol-Durak (2010)^8^ | Türkiye | Europe | 166 | 0.55 | Older Adults | Mixed | UCLA | General health | Perceived overall health | Subjective | -.3400 |
| 18 |  | Türkiye | Europe | 478 | 0.61 | Adults | Mixed | UCLA | General health | Perceived overall health | Subjective | -.2600 |
| 19 | Ebrecht et al. (2004)^9^ | United Kingdom | Europe | 24 | 0.00 | Adults | - | UCLA | General health | Speed of wound healing- ultrasound scan | Objective | -.0470 |
| 20 | Fauth et al. (2008)^10^ | Sweden | Europe | 140 | 0.70 | Older Adults | - | UCLA | Sensory | Vision rated by nurse | Subjective | -.2100 |
| 21 |  |  |  |  |  |  |  | UCLA | Sensory | Hearing rated by nurse | Subjective | -.1700 |
| 22 |  |  |  |  |  |  |  | UCLA | General health | Lung function- peak expiratory flow | Objective | -.2100 |
| 23 |  |  |  |  |  |  |  | UCLA | General health | Pulse per minute- pulse oximeter | Objective | -.1900 |
| 24 |  |  |  |  |  |  |  | UCLA | General health | Blood oxygen saturation- pulse oximeter | Objective | -.0200 |
| 25 |  |  |  |  |  |  |  | UCLA | Physical health | Grip strength- squeezing dynamometer | Objective | -.1200 |
| 26 |  |  |  |  |  |  |  | UCLA | Physical health | Functional limitations rated by nurse | Objective | -.2200 |
| 27 | Fernandez-Alonso et al. (2012)^11^ | Spain | Europe | 182 | 1.00 | Adults | Mixed | UCLA | General health | Self-reported systolic blood pressure | Subjective | -.0130 |
| 28 |  |  |  |  |  |  |  | UCLA | General health | Self-reported diastolic blood pressure | Subjective | -.0470 |
| 29 |  |  |  |  |  |  |  | UCLA | General health | Self-reported BMI | Subjective | -.1600 |
| 30 |  |  |  |  |  |  |  | UCLA | General health | Somatic Menopause Rating Scale (MRS) score (menopause symptoms) | Subjective | -.2420 |
| 31 |  |  |  |  |  |  |  | UCLA | General health | Urogenital MRS score (menopause symptoms) | Subjective | -.2150 |
| 32 | Finkenauer et al. (2002)^12^ | Netherlands | Europe | 227 | 0.51 | Children and Adolescents | - | UCLA | General health | Symptoms (nausea, tiredness) | Subjective | -.1940 |
| 33 | Freund & Baltes (1998)^13^ | Germany | Europe | 200 | 0.51 | Older Adults | - | UCLA | General health | Subjective overall health | Subjective | -.2150 |
| 34 | Gerstorf et al. (2006)^14^ | Germany | Europe | 130 | 0.55 | Older Adults | - | UCLA | Sensory | Auditory and visual accuracy composite | Objective | -.2050 |
| 35 | Hansson et al. (1987)^15^ | United States | North America | 102 | 0.65 | Older Adults | - | UCLA | General health | How often they became ill | Subjective | -.3200 |
| 36 | Hawkley et al. (2010)^16^ | United States | North America | 214 | 0.52 | Adults | Middle/High | UCLA | General health | BMI | Objective | -.0500 |
| 37 |  |  |  | 215 |  |  |  | UCLA | Sleep | Hours of sleep | Subjective | .0200 |
| 38 |  |  |  | 214 |  |  |  | UCLA | General health | Physical symptom severity (e.g. headaches) | Subjective | -.2900 |
| 39 | Hays & DiMatteo (1987)^17^ | United States | North America | 180 | 0.62 | Children and Adolescents | - | UCLA | Sleep | Hours of sleep | Subjective | 0 |
| 40 | Jaremka et al. (2013)^18^ | United States | North America | 200 | 1.00 | Adults | Middle/High | UCLA | General health | Self-reported BMI | Subjective | -.0800 |
| 41 |  |  |  |  |  |  |  | UCLA | Sleep | Sleep quality (sleep habits, sleep disturbance) | Subjective | -.2900 |
| 42 |  |  |  |  |  |  |  | UCLA | General health | Short Form health survey-36 (SF-36) pain and vitality subscales | Subjective | -.4500 |
| 43 |  |  |  | 156 | 1.00 | Adults | Middle/High | UCLA | General health | Epstein-Barr Virus (EBV) antibodies | Objective | .0600 |
| 44 |  |  |  | 82 | 1.00 | Adults | Middle/High | UCLA | General health | Cytomegalovirus (CMV) antibodies | Objective | -.2400 |
| 45 | Kahn et al. (2003)^19^ | United States | North America | 100 | 0.66 | Older Adults | - | UCLA | General health | Self-rated overall health | Subjective | .0700 |
| 46 | Kidd & Shahar (2008)^20^ | United States and Canada | North America | 208 | 0.40 | Children and Adolescents | - | UCLA | General health | Subjective health status | Subjective | -.2300 |
| 47 | Kuwert et al. (2014)^21^ | United States | North America | 2025 | 0.00 | Older Adults | - | UCLA | General health | BSI-18 somatization subscale | Subjective | -.2800 |
| 48 | Lawler-Row et al. (2011)^22^ | United States | North America | 114 | 0.55 | Children and Adolescents | - | UCLA | General health | Cohen-Hoberman Inventory of Physical Symptoms (CHIPS) scale | Subjective | -.4200 |
| 49 | Light & Visser (2013)^23^ | United States | North America | 1278 | 0.50 | Adults | - | UCLA | General health | Self-reported overall health | Subjective | -.2850 |
| 50 | Liu & Guo (2007)^24^ | China | Asia | 275 | 0.55 | Older Adults | Mixed | UCLA | Physical health | SF-36 physical functioning subscale | Subjective | -.1810 |
| 51 |  |  |  |  |  |  |  | UCLA | Physical health | SF-36 role limitations subscale | Subjective | -.2950 |
| 52 |  |  |  |  |  |  |  | UCLA | General health | SF-36 bodily pain subscale | Subjective | -0.2390 |
| 53 |  |  |  |  |  |  |  | UCLA | General health | SF-36 general health perceptions subscale | Subjective | -0.6480 |
| 54 |  |  |  |  |  |  |  | UCLA | General health | SF-36 vitality subscale | Subjective | -0.6670 |
| 55 |  | China | Asia | 315 | 0.55 | Older Adults | Mixed | UCLA | Physical health | SF-36 physical functioning subscale | Subjective | -.2880 |
| 56 |  |  |  |  |  |  |  | UCLA | Physical health | SF-36 role limitations subscale | Subjective | -.2630 |
| 57 |  |  |  |  |  |  |  | UCLA | General health | SF-36 bodily pain subscale | Subjective | -0.2460 |
| 58 |  |  |  |  |  |  |  | UCLA | General health | SF-36 general health perceptions subscale | Subjective | -0.6030 |
| 59 |  |  |  |  |  |  |  | UCLA | General health | SF-36 vitality subscale | Subjective | -0.5740 |
| 60 | Long & Martin (2000)^25^ | United States | North America | 100 | 0.77 | Older Adults | Mixed | UCLA | General health | Subjective overall health | Subjective | -.3400 |
| 61 |  | United States | North America | 100 | 0.55 | Adults | Middle/High | UCLA | General health | Subjective overall health | Subjective | -.3200 |
| 62 | Lutgendorf et al. (2004)^26^ | United States | North America | 554 | 0.61 | Older Adults | - | UCLA | General health | Blood- Interleukin 6 (IL-6) | Objective | -.0300 |
| 63 | Mahon (1994)^27^ | United States | North America | 106 | 0.51 | Children and Adolescents | - | UCLA | Sleep | sleep disturbance | Subjective | -0.1940 |
| 64 |  |  |  |  |  |  |  | UCLA | Sleep | sleep effectiveness | Subjective | -0.1430 |
| 65 |  |  |  |  |  |  |  | UCLA | Sleep | hours of sleep | Subjective | 0.0220 |
| 66 |  | United States | North America | 111 | 0.55 | Children and Adolescents | - | UCLA | Sleep | sleep disturbance | Subjective | -0.1980 |
| 67 |  |  |  |  |  |  |  | UCLA | Sleep | sleep effectiveness | Subjective | -0.1480 |
| 68 |  |  |  |  |  |  |  | UCLA | Sleep | hours of sleep | Subjective | 0.1140 |
| 69 |  | United States | North America | 113 | 0.50 | Children and Adolescents | - | UCLA | Sleep | sleep disturbance | Subjective | -0.1660 |
| 70 |  |  |  |  |  |  |  | UCLA | Sleep | sleep effectiveness | Subjective | -0.1420 |
| 71 |  |  |  |  |  |  |  | UCLA | Sleep | hours of sleep | Subjective | 0.0760 |
| 72 | Mahon et al. (2003)^28^ | United States | North America | 135 | 0.55 | Children and Adolescents | - | UCLA | General health | General health rating index | Subjective | -.4600 |
| 73 | Mahon et al. (1997)^29^ | United States | North America | 69 | 0.39 | Adults | - | UCLA | General health | General health rating index | Subjective | -.3500 |
| 74 | Margrett et al. (2011)^30^ | United States | North America | 132 | 0.74 | Older Adults | - | UCLA | Physical health | Physical limitations- Activities of Daily Living (ADL) | Subjective | -.1100 |
| 75 | Matthews-Ewald & Zullig (2013)^31^ | United States | North America | 723 | 0.68 | Children and Adolescents | - | UCLA | General health | Health-related Quality of Life scale- self-perceived health | Subjective | -.2400 |
| 76 | McConnell et al. (2011)^32^ | United States | North America | 217 | 0.79 | Adults | Middle/High | UCLA | General health | Physical illness and symptom inventory e.g. headaches | Subjective | -.2700 |
| 77 | Nausheen et al. (2007)^33^ | United Kingdom | Europe | 23 | 1.00 | Adults | - | UCLA | General health | Systolic blood pressure | Objective | -.2900 |
| 78 |  |  |  |  |  |  |  | UCLA | General health | Diastolic blood pressure | Objective | -.1700 |
| 79 |  |  |  |  |  |  |  | UCLA | General health | Heart rate | Objective | 0 |
| 80 | Pereira et al. (2013)^34^ | United States | North America | 345 | 0.75 | Children and Adolescents | - | UCLA | General health | Rotterdam Symptoms Checklist- physical symptoms e.g. nausea, tiredness | Subjective | -.4180 |
| 81 | Plouffe & Jomphe-Hill (1996)^35^ | Canada | North America | 97 | 0.84 | Older Adults | Low | UCLA | General health | Overall health | Subjective | -.2600 |
| 82 |  |  |  |  |  |  |  | UCLA | Physical health | Physical limitations | Subjective | -.0370 |
| 83 |  |  |  |  |  |  |  | UCLA | Use of services | GP visits | Subjective | .0110 |
| 84 |  |  |  |  |  |  |  | UCLA | General health | How many days sick | Subjective | -.2100 |
| 85 |  |  |  |  |  |  |  | UCLA | General health | Drugs to control pain | Subjective | -.1900 |
| 86 |  |  |  |  |  |  |  | UCLA | Sleep | Drugs to sleep | Subjective | -.2000 |
| 87 | Poulin et al. (2012)^36^ | United States and China | North America and Asia | 295 | 0.68 | Older Adults | Low | UCLA | Physical health | Functional health scale (A Guttman Health Scale for the Aged) | Subjective | -.3280 |
| 88 | Reichl et al. (2013)^37^ | Germany | Europe | 559 | 0.59 | Adults | - | UCLA | General health | Short Form health survey-12 (SF-12) physical health scale | Subjective | -.2100 |
| 89 | Reis et al. (1985)^38^ | United States | North America | 30 | 0 | Adults | - | UCLA | Use of services | Accidents | Objective | .2600 |
| 90 | Scott et al. (2011)^39^ | United States | North America | 282 | 0.61 | Older Adults | Mixed | UCLA | General health | Somatic health complaints e.g. headaches, joint stiffness | Subjective | -.2900 |
| 91 | Scott et al. (2013)^40^ | United States | North America | 410 | 0.59 | Adults | Mixed | UCLA | General health | Somatic health complaints e.g. headaches, joint stiffness | Subjective | -.2200 |
| 92 | Segrin & Domschke (2011)^41^ | United States | North America | 224 | 0.65 | Adults | - | UCLA | General health | Overall health self-rating | Subjective | -.2900 |
| 93 |  |  |  |  |  |  |  | UCLA | Sleep | Pittsburgh sleep quality index (PSQI) | Subjective | -.3500 |
| 94 |  |  |  |  |  |  |  | UCLA | General health | SF-36 general health quality of life subscale | Subjective | -.2500 |
| 95 | Segrin & Passalacqua (2010)^42^ | United States | North America | 265 | 0.52 | Adults | - | UCLA | General health | SF-36 general health quality of life subscale | Subjective | -.2200 |
| 96 |  |  |  |  |  |  |  | UCLA | Sleep | Adequate amount of sleep | Subjective | -.2400 |
| 97 | Smith et al. (2012)^43^ | Australia | Oceania | 97 | 0.71 | Adults | - | UCLA | Sleep | PSQI | Subjective | -.2900 |
| 98 | Springer et al. (2003)^44^ | United States | North America | 118 | 0.84 | Older Adults | - | UCLA | Physical health | Presence of any physical impariments | Subjective | -.2700 |
| 99 | Staight & Harvey (1990)^45^ | United States | North America | 25 | 1.00 | Older Adults | - | UCLA | General health | Overall health | Subjective | -.3460 |
| 100 | Sun et al. (2012)^46^ | United States | North America | 734 | 0.62 | Older Adults | Middle/High | UCLA | General health | Self-rated health (single item from SF-36) | Subjective | -.1900 |
| 101 | Utz et al. (2011)^47^ | United States | North America | 328 | 0.59 | Older Adults | Mixed | UCLA | Physical health | Meeting physical demands | Subjective | -.1700 |
| 102 | Walker & Beauchene (1991)^48^ | United States | North America | 58 | 0.88 | Older Adults | - | UCLA | Physical health | A Guttman health scale for the aged- 6 items on functional ability to perform daily tasks | Subjective | -.0500 |
| 103 | Wen et al. (2006)^49^ | United States | North America | 214 | 0.52 | Adults | Mixed | UCLA | General health | Self-rated health (single item from SF-36) | Subjective | -.1580 |
| 104 | Xu et al. (2011)^50^ | China | Asia | 5226 | 0.44 | Children and Adolescents | - | UCLA | General health | Self-rated health measurement scale | Subjective | -.6400 |
| 105 | Xu et al. (2012)^51^ | China | Asia | 5226 | 0.44 | Children and Adolescents | - | UCLA | Sleep | PSQI | Subjective | -.2530 |
| 106 | Alpass & Neville (2003)^52^ | New Zealand | Oceania | 217 | 0.00 | Older Adults | - | UCLA | General health | Self-rated health status | Subjective | -.3000 |
| 107 | Habersaat et al. (2015)^53^ | Switzerland | Europe | 84 | 0.19 | Adults | - | UCLA | General health | CHIPS scale | Subjective | -.1390 |
| 108 | Tse et al. (2013)^54^ | Hong Kong | Asia | 139 | 0.51 | Older Adults | Low | UCLA | Sleep | Hours of sleep | Subjective | .1930 |
| 109 |  |  |  |  |  |  |  | UCLA | Physical health | Barthel Index- ADL | Subjective | -.1330 |
| 110 |  |  |  |  |  |  |  | UCLA | Physical health | Elderly Mobility Scale | Subjective | -.0710 |
| 111 | Wagner et al. (2015)^55^ | Germany | Europe | 462 | 0.49 | Older Adults | - | UCLA | Physical health | Assistance required in ADL | Subjective | -.1200 |
| 112 | Nishina et al. (2005)^56^ | United States | North America | 1516 | 0.55 | Children and Adolescents | Low | Other | General health | Frequency of physical symptoms (e.g. headaches, nausea) | Subjective | -.2880 |
| 113 | Carcedo et al. (2011)^57^ | Spain | Europe | 140 | 0.50 | Adults | - | Other | Physical health | World Health Organisation Quality of Life short form survey (WHOQOL-BREF)- physical health | Subjective | -.1350 |
| 114 |  |  |  |  |  |  | ~~-~~ | Other | General health | WHOQOL-BREF- overall perception of health | Subjective | -.1780 |
| 115 | Bielderman et al. (2013)^58^ | Netherlands | Europe | 119 | 0.71 | Older Adults | Mixed | RTLS | Physical health | Groningen Frailty Indicator (GFI)- daily activities subscale | Subjective | .0030 |
| 116 |  |  |  |  |  |  |  | RTLS | General health | Groningen Frailty Indicator- health problems subscale | Subjective | -.3670 |
| 117 | Burke et al. (2012)^59^ | Ireland | Europe | 492 | 0.68 | Older Adults | - | RTLS | General health | Self-rated health status | Subjective | -.1800 |
| 118 | De Donder et al. (2012)^60^ | Belgium | Europe | 22010 | 0.49 | Older Adults | Mixed | RTLS | Physical health | Single-item physical health | Subjective | -.1980 |
| 119 |  |  |  |  |  |  |  | RTLS | Physical health | Need for mobility assistance | Subjective | -.1510 |
| 120 |  | Belgium | Europe | 4106 | 0.62 | Older Adults | Mixed | RTLS | Physical health | Single-item physical health | Subjective | -.2320 |
| 121 |  |  |  |  |  |  |  | RTLS | Physical health | Need for mobility assistance | Subjective | -.1700 |
| 122 | De Jong Gierveld & Van Tilburg (2006)^61^ | Netherlands | Europe | 7244 | 0.51 | Adults | - | RTLS | General health | Perceived overall health | Subjective | -.2200 |
| 123 |  | Netherlands | Europe | 3260 | 0.51 | Adults | - | RTLS | General health | Perceived overall health | Subjective | -.2300 |
| 124 |  | Netherlands | Europe | 2945 | 0.51 | Adults | - | RTLS | General health | Perceived overall health | Subjective | -.2400 |
| 125 | Eisses et al. (2004)^62^ | Netherlands | Europe | 393 | 0.75 | Older Adults | - | RTLS | General health | Nottingham Health Profile (NHP)- pain subscale | Subjective | -.1360 |
| 126 | . |  |  |  |  |  |  | RTLS | Physical health | Groningen Activity Restriction Scale (GARS)- functional impairment in ADL | Subjective | -.1860 |
| 127 | Iecovich (2013)^63^ | Israel | Asia | 2015 | 0.63 | Older Adults | Middle/High | RTLS | Physical health | Physical limitations-ADL | Subjective | -.3600 |
| 128 |  |  |  |  |  |  |  | RTLS | Physical health | Physical limitations-IADL | Subjective | -.4000 |
| 129 |  |  |  |  |  |  |  | RTLS | General health | Self-rated health | Subjective | -.4600 |
| 130 | Newall et al. (2009)^64^ | Canada | North America | 1243 | 0.50 | Older Adults | Mixed | RTLS | General health | Self-ratedhealth | Subjective | -.1900 |
| 131 |  |  |  |  |  |  |  | RTLS | Physical health | Independent functioning in ADL | Subjective | -.1700 |
| 132 | Sadler et al. (2006)^65^ | Netherlands | Europe | 1845 | 0.52 | Older Adults | - | RTLS | Physical health | Functional limitations | Subjective | -.2200 |
| 133 | Schnittger et al. (2012)^66^ | Ireland | Europe | 579 | 0.69 | Older Adults | - | RTLS | Physical health | Berg balance score | Obbjective | -.1540 |
| 134 |  |  |  |  |  |  |  | RTLS | General health | Pain (verbal rating scale) | Subjective | -.1290 |
| 135 |  |  |  |  |  |  |  | RTLS | General health | Recovery from illness (based on blood pressure) | Obbjective | -.0690 |
| 136 |  |  |  |  |  |  |  | RTLS | Physical health | Physical limitations- self-rated ADL | Subjective | -.1010 |
| 137 |  |  |  |  |  |  |  | RTLS | Physical health | Physical limitations- self-rated IADL | Subjective | -.1260 |
| 138 |  |  |  |  |  |  |  | RTLS | Sensory | Vision acuity | Objective | -.0760 |
| 139 |  |  |  |  |  |  |  | RTLS | General health | Baseline systolic blood pressure | Objective | .0480 |
| 140 |  |  |  |  |  |  |  | RTLS | General health | Maximum systolic blood pressure | Objective | .0210 |
| 141 |  |  |  |  |  |  |  | RTLS | General health | Nadir systolic blood pressure | Objective | .0006 |
| 142 |  |  |  |  |  |  |  | RTLS | General health | Delta systolic blood pressure | Objective | .0580 |
| 143 |  |  |  |  |  |  |  | RTLS | Sensory | Hearing test | Objective | -.0630 |
| 144 |  |  |  |  |  |  |  | RTLS | Physical health | Fried frailty index | Objective | -0.0710 |
| 145 |  |  |  |  |  |  |  | RTLS | Physical health | History of falls | Subjective | -0.1120 |
| 146 |  |  |  |  |  |  |  | RTLS | Sleep | PSQI | Subjective | -0.1440 |
| 147 | Steverink & Lindenberg (2008)^67^ | Netherlands | Europe | 439 | 0.58 | Older Adults | Mixed | RTLS | Physical health | Physical functioning- problems in ADL and IADL | Subjective | -.2500 |
| 148 | Van der Hal-Van Raalte et al. (2007)^68^ | Israel | Asia | 201 | 0.63 | Adults | - | RTLS | General health | Self-reported health | Subjective | -.3000 |
| 149 | Warner et al. (2010)^69^ | Germany | Europe | 1415 | 0.51 | Adults | - | RTLS | Physical health | SF-36 physical functioning subscale | Subjective | -.0800 |
| 150 |  |  |  |  |  |  |  | RTLS | Physical health | WHOQOL-BREF physical health subscale | Subjective | -.1900 |
| 151 | De Jong Gierveld & Van Tilburg (1999)^70^ | Italy | Europe | 1548 | 0.52 | Older Adults | - | RTLS | General health | Perceived overall health | Subjective | -.2300 |
| 152 | Dahlberg & Mckee (2014)^71^ | United Kingdom | Europe | 1255 | 0.62 | Older Adults | Low | RTLS | Physical health | Groningen Activity Restriction Scale | Subjective | -.2550 |
| 153 |  |  |  |  |  |  |  | RTLS | General health | Self-rated health | Subjective | -.2300 |
| 154 | Russell (1996)^72^ | United States | North America | 301 | 0.58 | Older Adults | - | UCLA | General health | Self-rated health | Subjective | -.1800 |
| 155 |  |  |  |  |  |  |  | UCLA | General health | Self-reported number of prescription medications | Subjective | -.0500 |
| 156 |  | United States | North America | 288 | 0.58 | Older Adults | - | UCLA | Physical health | Dukr-UNC Health Profile- physical functioning subscale | Subjective | -.0500 |
| 157 | Steed et al. (2007)^73^ | Australia | Oceania | 353 | 0.47 | Older Adults | Mixed | UCLA | General health | Self-rated health | Subjective | -.2600 |
| 158 |  |  |  |  |  |  |  | RTLS | General health | Self-rated health | Subjective | -.2700 |
| 159 | Becker (2014)^74^ | United States | North America | 126 | 0.54 | Children and Adolescents | Mixed | Other | Sleep | Children’s sleep habits questionnaire (CSHQ) | Subjective | -.3700 |
| 160 | Chen et al. (2014)^75^ | China | Asia | 521 | 0.66 | Older Adults | Mixed | UCLA | General health | Older people’s quality of life questionnaire- health domain | Subjective | -.4500 |
| 161 | Gerich (2014)^76^ | Austria | Europe | 246 | 0.48 | Adults | - | UCLA | General health | Single-item self-rated health | Subjective | -.2300 |
| 162 | Schumaker et al. (1985)^77^ | United States | North America | 132 | 0.51 | Adults | Mixed | UCLA | General health | Self-reported body weight | Subjective | -.2500 |
| 163 | Hartung & Renner (2014)^78^ | Germany | Europe | 77 | 0.81 | Adults | - | UCLA | General health | How many days sick | Subjective | -.3100 |
| 164 | Balter et al. (2019)^79^ | United Kingdom | Europe | 36 | 0.00 | Adults | - | UCLA | General health | IL-6 (immune dysregulation) | Objective | -.1230 |
| 165 | Bowen & Luy (2018)^80^ | Germany and Austria | Europe | 1041 | 0.59 | Older Adults | - | RTLS | General health | Self-rated health | Subjective | -.2100 |
| 166 | Cimarolli et al. (2018)^81^ | United States | North America | 119 | 0.78 | Older Adults | - | UCLA | Physical health | Functional disability- difficulties performing daily activities | Subjective | -.4200 |
| 167 |  |  |  |  |  |  |  | UCLA | Physical health | Health restricting participation in things they want to do | Subjective | -.1700 |
| 168 |  |  |  |  |  |  |  | UCLA | Sensory | Self-rated vision impairment | Subjective | -.2500 |
| 169 |  |  |  |  |  |  |  | UCLA | Sensory | Self-rated hearing impairment | Subjective | -.2400 |
| 170 | Corona et al. (2017)^82^ | United States | North America | 811 | 0.77 | Children and Adolescents | - | UCLA | General health | Self-rated health | Subjective | -.2900 |
| 171 |  |  |  |  |  |  |  | UCLA | General health | Cohen-Hoberman inventory of physical symptoms | Subjective | -.2900 |
| 172 | Corona et al. (2020)^83^ | United States | North America | 121 | 0.84 | Children and Adolescents | - | UCLA | General health | Single-item self-rated health | Subjective | -.5200 |
| 173 |  | United States | North America | 91 | 0.87 | Children and Adolescents | - | UCLA | General health | Single-item self-rated health | Subjective | -.3000 |
| 174 | Gonyea et al. (2016)^84^ | United States | North America | 216 | 0.73 | Older Adults | Low | UCLA | Physical health | Older Americans Resources and Services (OARS)- ADL and IADL scales | Subjective | -.2500 |
| 175 |  |  |  |  |  |  |  | UCLA | General health | Self-rated health | Subjective | -.2300 |
| 176 | Jung & Luck-Sikorski (2019)^85^ | Germany | Europe | 245 | 0.45 | Adults | - | UCLA | General health | Self-reported BMI | Subjective | -.1390 |
| 177 |  | Germany | Europe | 84 | 0.45 | Adults | - | UCLA | General health | Self-reported BMI | Subjective | -.1370 |
| 178 |  | Germany | Europe | 1000 | 0.45 | Adults | - | UCLA | General health | Self-rated health | Subjective | -.0070 |
| 179 | Lee et al. (2020)^86^ | United States | North America | 163 | 0.85 | Children and Adolescents | - | UCLA | General health | Single-item self-rated health | Subjective | -.2700 |
| 180 | Lin et al. (2016)^87^ | Australia | Oceania | 119 | 0.68 | Older Adults | - | RTLS | General health | Self-rated health | Subjective | -.4000 |
| 181 | Mund & Neyer (2016)^88^ | Germany | Europe | 654 | 0.54 | Adults | - | RTLS | General health | Single-item self-rated health | Subjective | -.1800 |
| 182 | Schutter et al. (2017)^89^ | Netherlands | Europe | 426 | 0.63 | Older Adults | - | RTLS | General health | Morning cortisol | Objective | -.0370 |
| 183 |  |  |  |  |  |  |  | RTLS | General health | Cortisol- area under the curve with respect to ground | Objective | -.0600 |
| 184 |  |  |  |  |  |  |  | RTLS | General health | Cortisol- area under the curve with respect to increase | Objective | .0020 |
| 185 | Steel et al. (2019)^90^ | United States | North America | 104 | 0.77 | Adults | - | UCLA | General health | Metabolic syndrome | Objective | -.0081 |
| 186 | Tan et al. (2020)^91^ | United Kingdom | Europe | 528 | 0.61 | Older Adults | - | RTLS | General health | SF-12 (health-related quality of life)- physical component summary | Subjective | -.9020 |
| 187 |  | Greece | Europe | 331 | 0.61 | Older Adults | - | RTLS | General health | SF-12 (health-related quality of life)- physical component summary | Subjective | -.3047 |
| 188 |  | Croatia | Europe | 481 | 0.61 | Older Adults | - | RTLS | General health | SF-12 (health-related quality of life)- physical component summary | Subjective | -.8833 |
| 189 |  | Netherlands | Europe | 336 | 0.61 | Older Adults | - | RTLS | General health | SF-12 (health-related quality of life)- physical component summary | Subjective | -.8394 |
| 190 | Tully et al. (2019)^92^ | Denmark, Spain, Germany and United Kingdom | Europe | 1281 | 0.62 | Older Adults | - | RTLS | General health | BMI | Objective | -.4181 |
| 191 | Yang et al. (2020)^93^ | China | Asia | 475 | 0.44 | Children and Adolescents | Mixed | Other | Sleep | PSQI | Subjective | -.3600 |
| 192 | Burns et al. (2022)^94^ | Ireland | Europe | 6671 | 0.54 | Adults | - | UCLA | Use of services | GP visits | Subjective | -.0697 |
| 193 |  |  |  | 6675 | 0.54 | Adults | - | UCLA | Use of services | Emergency department visits | Subjective | -0.0530 |
| 194 | Hajek & König (2021)^95^ | Germany | Europe | 3075 | 0.51 | Adults | - | RTLS | General health | Oral Health Impact Profile/ oral health-related quality of life | Subjective | -.2300 |
| 195 | Ho et al. (2021)^96^ | Vietnam | Asia | 354 | 0.54 | Children and Adolescents | - | UCLA | Sleep | PSQI | Subjective | -.4200 |
| 196 | Kino et al. (2023)^97^ | Japan | Asia | 13766 | 0.44 | Older Adults | Mixed | UCLA | Physical health | Instrumental activities of daily living (i) using public transportation, (ii) shopping for daily necessities, (iii) boiling water, (iv) paying bills, and (v) handling banking deposits) | Subjective | -.6474 |
| 197 | Hajek & König (2022)^98^ | Germany | Europe | 3075 | 0.51 | Adults | - | RTLS | General health | Single-item self-rated health | Subjective | -.1700 |
| 198 | Hisata et al. (2023)^99^ | Japan | Asia | 2700 | 0.55 | Older Adults | Mixed | UCLA | General health | Self-rated health | Subjective | -.1578 |
| 199 | Kim & Lee (2022)^100^ | South Korea | Asia | 1000 | 0.59 | Older Adults | Mixed | UCLA | General health | Self-rated health | Subjective | -.0200 |
| 200 |  |  |  |  |  |  |  | UCLA | Physical health | IADL scale | Subjective | -.0100 |
| 201 | Gyasi et al. (2022a)^101^ | Ghana | Africa | 1201 | 0.63 | Older Adults | - | UCLA | Sleep | Impaired sleep- sleep quality scale | Subjective | -.1730 |
| 202 | Gyasi et al. (2022b)^102^ | Ghana | Africa | 1201 | 0.63 | Older Adults | - | UCLA | Physical health | Physical function impairment scale | Subjective | -.2340 |
| 203 | Hussein et al. (2021)^103^ | Malaysia | Asia | 380 | 0.62 | Older Adults | Mixed | Other | General health | Single-item self-rated health | Subjective | -.2480 |
| 204 |  |  |  |  |  |  |  | Other | General health | RAND Short Form-20 (SF-20)- physical health deteriorated or not (reference group = yes) | Subjective | -.1470 |
| 205 | Lu et al. (2023)^104^ | Canada | North America | 968 | 0.52 | Older Adults | Low | UCLA | Use of services | Number of visits to the GP in the past year | Objective | -.0028 |
| 206 |  | Canada | North America | 1703 | 0.55 | Older Adults | Middle/High | UCLA | Use of services | Number of visits to the GP in the past year | Objective | -.0055 |
| 207 |  | Canada | North America | 968 | 0.52 | Older Adults | Low | UCLA | General health | Self-rated health | Subjective | -.1672 |
| 208 |  | Canada | North America | 1703 | 0.55 | Older Adults | Middle/High | UCLA | General health | Self-rated health | Subjective | -.0547 |
| 209 | Ozcan et al. (2022)^105^ | Türkiye | Europe | 197 | 1.00 | Adults | Mixed | UCLA | General health | MENQOL (health-orientated quality of life during menopausal period scale)- vasomotor symptoms subscale | Subjective | -.2590 |
| 210 |  |  |  |  |  |  |  | UCLA | General health | MENQOL- physical symptoms subscale | Subjective | -.4050 |
| 211 | Nguyen et al. (2021)^106^ | United States | North America | 184 | 0.48 | Adults | - | UCLA | General health | Gut microbiome (faecal sample; BD SWUBE Dual Swab Collection System) | Objective | -.1700 |
| 212 | Meisters et al. (2021)^107^ | Netherlands | Europe | 114,428 | 0.53 | Adults | Mixed | RTLS | Use of services | GP expenditure | Objective | -.1480 |
| 213 |  |  |  |  |  |  |  | RTLS | Use of services | Pharmaceutical expenditure | Objective | -.3020 |
| 214 | Mead et al. (2023)^108^ | United States | North America | 7749 | 0.58 | Older Adults | Mixed | UCLA | Sleep | Self-reported sleep health- four items | Subjective | -.1220 |
| 215 | Masaeli & Farhadi (2021)^109^ | Iran | Asia | 298 | 0.55 | Adults | - | UCLA | Sleep | Quality of sleep scale | Subjective | -.1800 |
| 216 | Lutzman et al. (2021)^110^ | Israel | Asia | 198 | 0.00 | Older Adults | - | UCLA | General health | Physical pain subscale from WHO Study on global AGEing and adult health | Subjective | -.3300 |
| 217 | Luo & Hu (2022)^111^ | China | Asia | 487 | 0.42 | Children and Adolescents | Mixed | UCLA | Sleep | PSQI | Subjective | -.2940 |
| 218 | Loughrey et al. (2021)^112^ | Ireland | Europe | 66 | 0.68 | Older Adults | - | RTLS | Sensory | Hearing- pure-tone air conduction decibel thresholds | Objective | -.1372 |
| 219 | Littlejohn et al. (2022)^113^ | United Kingdom | Europe | 80 | 0.66 | Older Adults | Mixed | RTLS | Sensory | Hearing difficulties- Speech, Spatial and Qualities of Hearing scale | Subjective | -.1890 |
| 220 | Lin et al. (2022)^114^ | Taiwan | Asia | 53 | 0.49 | Older Adults | - | UCLA | Physical health | Pittsburgh Fatigability Scale- physical score | Subjective | -.2800 |
| 221 | Kuang et al. (2023)^115^ | United States | North America | 2624 | 0.54 | Older Adults | - | UCLA | Physical health | Gait speed (timed 3-m walk: unable to walk, ≥5.7 s, and <5.7 s) | Objective | -.0390 |
| 222 |  |  |  |  |  |  |  | UCLA | Physical health | Self-reported difficulty walking one block | Subjective | -.1940 |
| 223 |  |  |  |  |  |  |  | UCLA | Physical health | Self-reported difficulty walking across room | Subjective | -.0386 |
| 224 | Krobisch et al. (2021)^116^ | Germany | Europe | 194 | 0.48 | Older Adults | Mixed | RTLS | General health | Self-rated health | Subjective | -.0570 |
| 225 | Killgore et al. (2022)^117^ | United States | North America | 2061 | 0.56 | Adults | - | UCLA | Sleep | Insomnia Severity Index (ISI) | Subjective | -.0980 |
| 226 | Jopling et al. (2021)^118^ | Canada | North America | 52 | 0.40 | Children and Adolescents | - | UCLA | General health | Chemiluminescence immunoassay (Cortisol at waking) | Objective | .0490 |
| 227 | Johar et al. (2021)^119^ | Germany | Europe | 500 | 0.37 | Older Adults | - | UCLA | General health | Cortisol awakening response (CAR) | Objective | -.0300 |
| 228 |  |  |  |  |  |  |  | UCLA | General health | Late night salivary cortisol | Objective | -.0020 |
| 229 |  |  |  |  |  |  |  | UCLA | General health | Diurnal cortisol slope | Objective | -.2000 |
| 230 | Jiang et al. (2021)^120^ | China | Asia | 7070 | 0.60 | Older Adults | Middle/high | UCLA | Sensory | Single-item self-reported hearing impairment | Subjective | -.0240 |
| 231 |  |  |  |  |  |  |  | UCLA | Physical health | Self-reported difficulty in basic activities of daily living | Subjective | -.0030 |
| 232 |  |  |  |  |  |  |  | UCLA | Sensory | Self-reported hearing aid use | Subjective | -.0270 |
| 233 | Jiang et al. (2022)^121^ | China | Asia | 3769 | 0.63 | Older Adults | Mixed | UCLA | Sensory | Pure-tone audiometry test | Objective | -.0500 |
| 234 |  |  |  |  |  |  |  | UCLA | Sensory | Self-reported hearing aid usage | Subjective | -.0240 |
| 235 |  |  |  |  |  |  |  | UCLA | Physical health | Self-reported difficulty in basic activities of daily living | Subjective | -.0110 |
| 236 | Huang et al. (2021)^122^ | United States | North America | 3196 | 0.53 | Older Adults | Mixed | UCLA | General health | Self-rated health | Subjective | -.1730 |
| 237 |  |  |  |  |  |  |  | UCLA | Sensory | Individual perception of functional hearing impairment | Subjective | -.1110 |
| 238 |  |  |  |  |  |  |  | UCLA | Physical health | Number of IADLs done with difficulty | Subjective | -.0360 |
| 239 |  |  |  |  |  |  |  | UCLA | Physical health | ADL impairments | Subjective | -.0110 |
| 240 | Hofman et al. (2022)^123^ | Netherlands | Europe | 7885 | 0.57 | Adults | Mixed | RTLS | General health | Single-item self-perceived health | Subjective | -.0450 |
| 241 |  |  |  |  |  |  |  | RTLS | Physical health | Single-item self-reported functional limitations in daily life | Subjective | 0 |
| 242 |  |  |  |  |  |  |  | RTLS | General health | BMI (self-reported height and weight) | Subjective | -.0250 |
| 243 | Hawkley et al. (2022)^124^ | United States | North America | 2440 | 0.54 | Adults | Mixed | UCLA | General health | Single-item self-rated health | Subjective | -.2000 |
| 244 | Hafstad et al. (2022)^125^ | Norway | Europe | 3564 | 0.49 | Children and Adolescents | Middle/high | UCLA | General health | Children's Somatic Symptoms Inventory (CSSI) | Subjective | -.1180 |
| 245 | Abu Elheja et al. (2021)^126^ | Israel | Asia | 63 | 0.68 | Older Adults | - | RTLS | General health | Single-item self-rated health | Subjective | -.2700 |
| 246 | Albert (2021)^127^ | Luxembourg | Europe | 131 | 0.52 | Adults | - | UCLA | General health | Self-rated health | Subjective | -.2900 |
| 247 | Baumbach et al. (2023)^128^ | Germany | Europe | 4264 | 0.54 | Adults | - | RTLS | General health | Self-rated health | Subjective | -.2300 |
| 248 |  |  |  |  |  |  |  | RTLS | General health | BMI (self-reported height and weight) | Subjective | -.0800 |
| 249 |  |  |  |  |  |  |  | RTLS | Physical health | SF-36 physical functioning subscale | Subjective | -.1700 |
| 250 | Benson et al. (2021)^129^ | United States | North America | 759 | 0.53 | Older Adults | - | UCLA | Sleep | Single-item self-reported sleep duration | Subjective | -.1100 |
| 251 |  |  |  |  |  |  |  | UCLA | Sleep | Sleep log-calculated sleep duration | Subjective | -.0500 |
| 252 |  |  |  |  |  |  |  | UCLA | Sleep | NSHAP core survey calculated time in bed | Subjective | .0200 |
| 253 |  |  |  |  |  |  |  | UCLA | Sleep | Insomnia symptoms | Subjective | -.1500 |
| 254 |  |  |  |  |  |  |  | UCLA | Sleep | Actigraphy total sleep time | Objective | -.0500 |
| 255 |  |  |  |  |  |  |  | UCLA | Sleep | Actigraph wake after sleep onset (WASO) | Objective | -.0800 |
| 256 |  |  |  |  |  |  |  | UCLA | Sleep | Actigraph percent sleep | Objective | -.0700 |
| 257 | Bergman et al. (2021)^130^ | Israel | Asia | 1112 | 0.75 | Adults | - | UCLA | General health | Self-rated health | Subjective | -.0600 |
| 258 | Chang et al. (2022)^131^ | China | Asia | 1262 | 0.51 | Older Adults | - | UCLA | General health | Self-rated health | Subjective | -.6320 |
| 259 | Chong et al. (2022)^132^ | Singapore | Asia | 497 | 0.57 | Older Adults | - | UCLA | Physical health | Functional ability- daily living without assistance | Subjective | -.1000 |
| 260 |  |  |  |  |  |  |  | UCLA | Physical health | Functional ability- fall history in the past 6 months | Subjective | .0100 |
| 261 | Crespo-Sanmiguel et al. (2022)^133^ | Spain | Europe | 79 | 0.51 | Older Adults | - | UCLA | General health | World Health Organisation quality of life short form survey (physical health subscale) | Subjective | -.2340 |
| 262 |  |  |  |  |  |  |  | UCLA | General health | BMI | Objective | -.0440 |
| 263 |  |  |  |  |  |  |  | UCLA | General health | Cortisol awakening response | Objective | .0470 |
| 264 |  |  |  |  |  |  |  | UCLA | General health | Diurnal cortisol slope | Objective | -.0910 |
| 265 | Czaja et al. (2021)^134^ | United States | North America | 300 | 0.78 | Older Adults | Low | UCLA | General health | Single-item self-rated health | Subjective | -.3500 |
| 266 |  |  |  |  |  |  |  | UCLA | Physical health | Life Space Questionnaire- mobility in past 3 days | Subjective | -.4300 |
| 267 |  |  |  |  |  |  |  | UCLA | Physical health | Functional activity limitations | Subjective | -.8900 |
| 268 | Das et al. (2021)^135^ | Bangladesh | Asia | 672 | 0.43 | Adults | - | UCLA | Sleep | PSQI | Subjective | -.2580 |
| 269 |  |  |  |  |  |  |  | UCLA | General health | Self-reported BMI | Subjective | -.1240 |
| 270 | Encarnação et al. (2023)^136^ | Portugal | Europe | 23 | 0.83 | Older Adults | - | UCLA | General health | Body fat percentage | Objective | -.4400 |
| 271 |  |  |  |  |  |  |  | UCLA | General health | Waist circumference | Objective | -.3000 |
| 272 |  |  |  |  |  |  |  | UCLA | General health | Hip circumference | Objective | -.4100 |
| 273 |  |  |  |  |  |  |  | UCLA | Physical health | Hand grip strength | Objective | -.2200 |
| 274 |  |  |  |  |  |  |  | UCLA | Physical health | Upper limb strength- arm curl test | Objective | -.2400 |
| 275 |  |  |  |  |  |  |  | UCLA | Physical health | Lower limb power- five-time sit-to-stand test | Objective | .0500 |
| 276 |  |  |  |  |  |  |  | UCLA | Physical health | Lower limb muscle strength- seat-to-stand test | Objective | -.1900 |
| 277 |  |  |  |  |  |  |  | UCLA | Physical health | Dynamic balance- time-up-and-go test | Objective | .1400 |
| 278 |  |  |  |  |  |  |  | UCLA | Physical health | Aerobic fitness- two-minute step test | Objective | -.4900 |
| 279 |  |  |  |  |  |  |  | UCLA | General health | Lean mass | Objective | .3700 |
| 280 |  |  |  |  |  |  |  | UCLA | General health | Basal metabolism | Objective | -.3000 |
| 281 | Grossman et al. (2021)^137^ | Israel | Asia | 243 | 0.69 | Older Adults | - | UCLA | General health | Single-item self-rated health | Subjective | .0100 |
| 282 |  |  |  |  |  |  |  | UCLA | Sleep | Self-reported sleep problems | Subjective | -.3600 |
| 283 | Gu et al. (2021)^138^ | China | Asia | 289 | 0.72 | Adults | - | UCLA | Sleep | PSQI | Subjective | -.3330 |
| 284 | Xiao et al. (2022)^139^ | China | Asia | 3250 | - | Older Adults | - | UCLA | Sleep | PSQI | Subjective | -.2560 |
| 285 |  |  |  |  |  |  |  | UCLA | Physical health | Barthel Index- ADL | Subjective | -.3110 |
| 286 | Zilioli & Jiang (2021)^140^ | United States | North America | 314 | 0.54 | Adults | - | UCLA | General health | Salivary cortisol at awakening | Objective | 0 |
| 287 |  |  |  |  |  |  |  | UCLA | General health | Salivary cortisol awakening response | Objective | .0600 |
| 288 |  |  |  |  |  |  |  | UCLA | General health | Salivary cortisol diurnal slope | Objective | -.1300 |
| 289 |  |  |  |  |  |  |  | UCLA | General health | Systemic inflammation- CRP | Objective | -.1100 |
| 290 |  |  |  |  |  |  |  | UCLA | General health | Systemic inflammation- IL-6 | Objective | -.1500 |
| 291 |  |  |  |  |  |  |  | UCLA | General health | Adiposity- waist-hip ratio | Objective | -.0100 |
| 292 | Yu et al. (2021)^141^ | United States | North America | 4680 | 0.56 | Older Adults | - | UCLA | General health | Self-rated health | Subjective | -.2300 |
| 293 |  |  |  |  |  |  |  | UCLA | Sensory | Self-rated eyesight | Subjective | -.1900 |
| 294 |  |  |  |  |  |  |  | UCLA | Sensory | Self-rated hearing | Subjective | -.0900 |
| 295 |  |  |  |  |  |  |  | UCLA | General health | Pain | Subjective | -.1600 |
| 296 |  |  |  |  |  |  |  | UCLA | Physical health | Falls- number of times in past two years | Subjective | -.0700 |
| 297 |  |  |  |  |  |  |  | UCLA | General health | Urinary incontinence- number of days in past month | Subjective | -.1000 |
| 298 |  |  |  |  |  |  |  | UCLA | Physical health | ADL scale | Subjective | -.1600 |
| 299 |  |  |  |  |  |  |  | UCLA | General health | Hospitalisation- within past two years | Subjective | -.0500 |
| 300 | Rufarakh et al. (2021)^142^ | Pakistan | Asia | 500 | 0.51 | Adults | - | UCLA | Sleep | PROMIS sleep disturbance short form | Subjective | -.2600 |
| 301 | Van Bogart et al. (2022)^143^ | United States | North America | 222 | 0.63 | Older Adults | Mixed | UCLA | General health | Inflammatory markers in blood- basal cytokines | Objective | .0800 |
| 302 |  |  |  |  |  |  |  | UCLA | General health | Inflammatory markers in blood- stimulated cytokines | Objective | -.0200 |
| 303 |  |  |  |  |  |  |  | UCLA | General health | Inflammatory markers in blood- basal CRP | Objective | -.1200 |
| 304 | Yuan et al. (2022)^144^ | China | Asia | 822 | 0.57 | Older Adults | Mixed | UCLA | Physical health | Geriatric Locomotive Function Scale | Subjective | -.1700 |
| 305 | Stout et al. (2023)^145^ | United States | North America | 31 | 1.00 | Children and Adolescents | - | UCLA | General health | Salivary cortisol- area under the curve with respect to increase | Objective | .3600 |
| 306 |  |  |  |  |  |  |  | UCLA | General health | Baseline cortisol concentration | Objective | -.4100 |
| 307 | Yang et al. (2021)^146^ | Singapore | Asia | 69 | 0.77 | Older Adults | - | UCLA | General health | Self-rated health (single item from SF-36) | Subjective | -.1200 |
| 308 |  |  |  |  |  |  |  | UCLA | Physical health | SF-36 item- degree of limitation in practical activities in daily life | Subjective | .0200 |
| 309 | Pataka et al. (2022)^147^ | Greece | Europe | 469 | 0.53 | Adults | - | UCLA | Sleep | Sleep Condition Indicator (SCI) | Subjective | -.2800 |
| 310 |  |  |  |  |  |  |  | UCLA | Sleep | SCI | Subjective | -.4500 |
| 311 | Perez et al. (2022)^148^ | United States | North America | 2534 | 0.55 | Adults | - | UCLA | General health | Self-rated health | Subjective | -.2900 |
| 312 |  |  |  |  |  |  |  | UCLA | Sleep | Weekday sleep duration | Subjective | -.1000 |
| 313 |  |  |  |  |  |  |  | UCLA | Sleep | Weekend sleep duration | Subjective | -.1990 |
| 314 |  |  |  |  |  |  |  | UCLA | Sleep | Trouble sleeping | Subjective | -.3400 |
| 315 |  |  |  |  |  |  |  | UCLA | General health | BMI status (self-reported height and weight) | Subjective | 0 |
| 316 | Savci et al. (2021)^149^ | Türkiye | Europe | 103 | 0.20 | Older Adults | Middle/High | RTLS | Physical health | WHOQOL-BREF physical health subscale | Subjective | -.2330 |
| 317 | Tümer et al. (2022)^150^ | Türkiye | Europe | 291 | 0.54 | Older Adults | Mixed | RTLS | Physical health | Aging in Place Scale- physical competence subscale | Subjective | -.2360 |
| 318 | Takeda et al. (2023)^151^ | Japan | Asia | 907 | 1.00 | Children and Adolescents | - | UCLA | General health | Premenstrual symptoms | Subjective | -.3950 |
| 319 |  |  |  |  |  |  |  | UCLA | General health | Self-reported BMI | Subjective | -.0080 |
| 320 |  |  |  |  |  |  |  | UCLA | General health | Menstrual pain intensity | Subjective | -.0230 |
| 321 |  |  |  |  |  |  |  | UCLA | Sleep | Sleep time | Subjective | .0210 |
| 322 | Ten Kate et al. (2021)^152^ | Germany | Europe | 606 | 0.48 | Adults | - | RTLS | General health | Single-item self-rated health | Subjective | -.2600 |
| 323 | Tian & Wang (2023)^153^ | China | Asia | 459 | 0.57 | Older Adults | - | UCLA | Sleep | PSQI | Subjective | -.3700 |
| 324 | Turner-Cobb et al. (2022)^154^ | United Kingdom | Europe | 530 | 0.61 | Adults | Mixed | UCLA | General health | Self-rated health (single item from SF-36) | Subjective | -.3000 |
| 325 | Zwilling (2022)^155^ | Israel | Asia | 246 | 0.67 | Adults | - | UCLA | Sleep | Self-rated sleep hours | Subjective | -.0110 |
| 326 | Rumas et al. (2021)^156^ | United States and Canada | North America | 790 | 0.55 | Adults | Mixed | UCLA | Physical health | WHOQOL-BREF physical health subscale | Subjective | -.3200 |
| 327 | Zawadzki & Gavrilova (2021)^157^ | United States | North America | 114 | 0.81 | Children and Adolescents | - | UCLA | General health | Systolic blood pressure | Objective | -.0180 |
| 328 |  |  |  |  |  |  |  |  |  | Diastolic blood pressure |  | .0600 |
| 329 | Roddick & Chen (2021)^158^ | Canada | North America | 265 | 1.00 | Adults | - | UCLA | General health | Resting heart rate variability | Objective | -.2000 |
| 330 | Xu et al. (2021)^159^ | China | Asia | 1456 | 0.59 | Adults | Mixed | UCLA | General health | Patient Health Questionnaire (PHQ-15) somatic symptoms | Subjective | -.0450 |
| 331 |  |  |  |  |  |  |  | UCLA | General health | Number of regular medications | Objective | -.0870 |
| 332 |  |  |  |  |  |  |  | UCLA | General health | Self-rated health | Subjective | -.0990 |
| 333 | Sams et al. (2021)^160^ | United States | North America | 501 | 0.66 | Older Adults | Mixed | UCLA | General health | PROMIS Global Health scale | Subjective | -.3710 |
| 334 | Takács et al. (2023)^161^ | Hungary | Europe | 2508 | 0.57 | Children and Adolescents | Mixed | UCLA | General health | Self-reported BMI | Subjective | -.0260 |
| 335 |  |  |  |  |  |  |  | UCLA | General health | Self-rated health | Subjective | -.0720 |
| 336 |  |  |  |  |  |  |  | UCLA | Sleep | Self-rated sleep | Subjective | 0 |
| 337 | Straus et al. (2022)^162^ | United States | North America | 4069 | 0.10 | Adults | Mixed | UCLA | Sleep | Sleep disorder | Subjective | -.0530 |
| 338 |  |  |  |  |  |  |  | UCLA | Physical health | Self-reported ADL disability | Subjective | -.1070 |
| 339 |  |  |  |  |  |  |  | UCLA | Physical health | Self-reported IADL disability | Subjective | -.0850 |
| 340 | Wang et al. (2021)^163^ | China | Asia | 1591 | 1.00 | Adults | Mixed | UCLA | Sleep | PSQI | Subjective | -.1490 |

*Notes*. UCLA = University of California Los Angeles Loneliness Scale; RTLS = Rasch-Type Loneliness Scale. Effect sizes were revised to be in the same direction. Gender is represented as a continuous variable whereby values less than 0.50 reflect a great proportion of males and values greater than 0.50 reflect a greater proportion of females; values of 0.00 reflect fully male samples while values of 1.00 represent fully female samples.

**Table S2.** Moderator Analyses of the Relationships Between Loneliness and Health Domains

| Model | Moderator | *k* | *b* | *b SE* | 95%CI | *F*(df1,df2) | *p* |
| --- | --- | --- | --- | --- | --- | --- | --- |
| Global health | | | | | | | |
| 1 | Publication year | 340 | .0000 | .0016 | -.0031, .0032 | 0.0008 (1, 338) | .9769 |
| 2 | Publication year- outliers removed | 328 | .0012 | .0012 | -.0011, .0036 | 1.0303 (1, 332) | .3108 |
| 3 | Continent | 332 |  |  |  | 1.0262 (2, 329) | .3595 |
|  | Asia | 71 | -.2529 | .0306 | -.3131, -.1928 |  |  |
|  | Europe | 121 | -.2077 | .0254 | -.2577, -.1577 |  |  |
|  | North America | 140 | -.1996 | .0234 | -.2456, -.1536 |  |  |
| 4 | Continent- outliers removed | 326 |  |  |  | 1.3898 (2, 323) | .2506 |
|  | Asia | 69 | -.2306 | .0224 | -.2746, -.1866 |  |  |
|  | Europe | 111 | -.1847 | .0185 | -.2211, -.1483 |  |  |
|  | North America | 140 | -.1916 | .0171 | -.2252, -.1580 |  |  |
| 5 | Gender | 338 | -.0325 | .0823 | -.1943, .1293 | 0.1560 (1, 336) | .6931 |
| 6 | Gender- outliers removed | 332 | -.0334 | .0621 | -.1557, .0888 | 0.2892 (1, 330) | .5911 |
| 7 | Loneliness scale | 340 |  |  |  | 0.8488 (2, 337) | .4289 |
|  | Other | 7 | -.2714 | .0833 | -.4351, -.1076 |  |  |
|  | RTLS | 68 | -.2480 | .0316 | -.3102, -.1858 |  |  |
|  | UCLA | 265 | -.2078 | .0166 | -.2405, -.1751 |  |  |
| 8 | Loneliness scale- outliers removed | 334 |  |  |  | 0.8306 (2, 331) | .4367 |
|  | Other | 7 | -.2711 | .0612 | -.3914, -.1508 |  |  |
|  | RTLS | 65 | -.2119 | .0232 | -.2575, -.1664 |  |  |
|  | UCLA | 262 | -.1965 | .0123 | -.2208, -.1723 |  |  |
| 9 | Type of health outcome measurement | 340 |  |  |  | 17.0050 (1, 338) | **<.0001** |
|  | Objective | 74 | -.1203 | .0275 | -.1744, -.0662 |  |  |
|  | Subjective | 266 | -.2358 | .0147 | -.2647, -.2068 |  |  |
| 10 | Type of health outcome measurement - outliers removed | 334 |  |  |  | 24.5994 (1, 332) | **<.0001** |
|  | Objective | 74 | -.1144 | .0203 | -.1543, -.0745 |  |  |
|  | Subjective | 260 | -.2168 | .0107 | -.2378, -.1958 |  |  |
| 11 | Age category | 340 |  |  |  | 1.3499 (2, 337) | .2607 |
|  | Adults | 99 | -.1866 | .0244 | -.2346, -.1386 |  |  |
|  | Children and adolescents | 41 | -.2461 | .0390 | -.3229, -.1694 |  |  |
|  | Older adults | 200 | -.2314 | .0199 | -.2706, -.1923 |  |  |
| 12 | Age category- outliers removed | 334 |  |  |  | 1.0141 (2, 331) | .3638 |
|  | Adults | 99 | -.1855 | .0180 | -.2208, -.1502 |  |  |
|  | Children and adolescents | 40 | -.2326 | .0291 | -.2898, -.1754 |  |  |
|  | Older adults | 195 | -.2054 | .0148 | -.2345, -.1763 |  |  |
| 13 | Socioeconomic Status (SES) | 136 |  |  |  | 0.6642 (2, 133) | .5164 |
|  | Low SES | 22 | -.2617 | .0521 | -.3646, -.1587 |  |  |
|  | Middle/high SES | 28 | -.1873 | .0431 | -.2725, -.1021 |  |  |
|  | Mixed SES | 86 | -.2145 | .0256 | -.2652, -.1638 |  |  |
| 14 | SES- outliers removed | 134 |  |  |  | 0.3096 (2, 131) | .7343 |
|  | Low SES | 21 | -.2209 | .0415 | -.3029, -.1388 |  |  |
|  | Middle/high SES | 28 | -.1825 | .0336 | -.2490, -.1161 |  |  |
|  | Mixed SES | 85 | -.2029 | .0205 | -.2434, -.1625 |  |  |
| General health | | | | | | | |
| 15 | Publication year | 184 | .0027 | .0022 | -.0016, .0069 | 1.5344 (1, 182) | .2170 |
| 16 | Publication year- outliers removed | 180 | .0037 | .0016 | .0007, .0068 | 5.7362 (1, 178) | .0177 |
| 17 | Continent | 180 |  |  |  | 1.0220 (2, 177) | .3620 |
|  | Asia | 29 | -.2927 | .0473 | -.3859, -.1994 |  |  |
|  | Europe | 77 | -.2184 | .0318 | -.2812, -.1555 |  |  |
|  | North America | 74 | -.2181 | .0300 | -.2773, -.1588 |  |  |
| 18 | Continent- outliers removed | 176 |  |  |  | 2.4005 (2, 173) | .0937 |
|  | Asia | 28 | -.2674 | .0339 | -.3344, -.2005 |  |  |
|  | Europe | 74 | -.1790 | .0224 | -.2233, -.1348 |  |  |
|  | North America | 74 | -.2137 | .0213 | -.2557, -.1718 |  |  |
| 19 | Gender | 184 | -.0187 | .1018 | -.1822, .2197 | 0.0338 (1, 182) | .8543 |
| 20 | Gender- outliers removed | 180 | .0070 | .0751 | -.1411, .1552 | 0.0088 (1, 178) | .9256 |
| 21 | Loneliness scale | 180 |  |  |  | 1.3455 (1, 178) | .2476 |
|  | RTLS | 39 | -.2762 | .0410 | -.3572, -.1953 |  |  |
|  | UCLA | 141 | -.2223 | .0226 | -.2669, -.1776 |  |  |
| 22 | Loneliness scale- outliers removed | 176 |  |  |  | 0.0012 (1, 174) | .9720 |
|  | RTLS | 36 | -.2136 | .0299 | -.2727, -.1546 |  |  |
|  | UCLA | 140 | -.2124 | .0165 | -.2450, -.1799 |  |  |
| 23 | Type of health outcome measurement | 184 |  |  |  | 17.1201 (1, 182) | **<.0001** |
|  | Objective | 49 | -.0894 | .0394 | -.1671, -.0116 |  |  |
|  | Subjective | 135 | -.2649 | .0200 | -.3043, -.2255 |  |  |
| 24 | Type of health outcome measurement - outliers removed | 180 |  |  |  | 25.7529 (1, 179) | **<.0001** |
|  | Objective | 49 | -.0843 | .0284 | -.1403, -.0283 |  |  |
|  | Subjective | 131 | -.2411 | .0143 | -.2693, -.2130 |  |  |
| 25 | Age category | 184 |  |  |  | 1.4565 (2, 181) | .2358 |
|  | Adults | 69 | -.1927 | .0312 | -.2543, -.1312 |  |  |
|  | Children and adolescents | 24 | -.2652 | .0502 | -.3642, -.1661 |  |  |
|  | Older adults | 91 | -.2577 | .0279 | -.3127, -.2027 |  |  |
| 26 | Age category- outliers removed | 180 |  |  |  | 0.9341 (2, 177) | .3949 |
|  | Adults | 69 | -.1891 | .0225 | -.2335, -.1448 |  |  |
|  | Children and adolescents | 23 | -.2315 | .0376 | -.3058, -.1572 |  |  |
|  | Older adults | 88 | -.2269 | .0203 | -.2668, -.1869 |  |  |
| 27 | Socioeconomic Status (SES) | 68 |  |  |  | 0.2094 (2, 65) | .8116 |
|  | Low SES | 9 | -.2622 | .0604 | -.3829, -.1415 |  |  |
|  | Middle/high SES | 13 | -.2152 | .0526 | -.3202, -.1102 |  |  |
|  | Mixed SES | 46 | -.2237 | .0297 | -.2831, -.1644 |  |  |
| Physical health | | | | | | | |
| 28 | Publication year | 75 | -.0035 | .0036 | -.0107, .0036 | 0.9655 (1, 73) | .3290 |
| 29 | Publication year- outlier removed | 73 | -.0008 | .0022 | -.0052, .0036 | 0.1396 (1, 71) | .7098 |
| 30 | Continent | 73 |  |  |  | 0.6804 (2, 70) | .6804 |
|  | Asia | 20 | -.2199 | .0542 | -.3279, -.1119 |  |  |
|  | Europe | 29 | -.1611 | .0500 | -.2609, -.0614 |  |  |
|  | North America | 24 | -.2112 | .0494 | -.3096, -.1127 |  |  |
| 31 | Continent- outlier removed | 71 |  |  |  | 0.0534 (2, 68) | .9481 |
|  | Asia | 19 | -.1763 | .0344 | -.2450, -.1076 |  |  |
|  | Europe | 29 | -.1620 | .0306 | -.2230, -.1010 |  |  |
|  | North America | 23 | -.1721 | .0303 | -.2326, -.1116 |  |  |
| 32 | Gender | 74 | -.1403 | .1881 | -.5152, .2346 | 0.5565 (1, 72) | .4581 |
| 33 | Gender- outlier removed | 72 | -.1600 | .1136 | -.3866, .0665 | 1.9856 (1, 70) | .1632 |
| 34 | Loneliness scale | 74 |  |  |  | 0.1112 (1, 72) | .7397 |
|  | RTLS | 22 | -.1865 | .0527 | -.2917, -.0814 |  |  |
|  | UCLA | 52 | -.2075 | .0345 | -.2763, -.1388 |  |  |
| 35 | Loneliness scale- outlier removed | 72 |  |  |  | 0.2204 (1, 70) | .6402 |
|  | RTLS | 22 | -.1878 | .0316 | -.2508, -.1248 |  |  |
|  | UCLA | 50 | -.1698 | .0217 | -.2131, -.1265 |  |  |
| 36 | Type of health outcome measurement | 75 |  |  |  | 0.3323 (1, 73) | .5661 |
|  | Objective | 10 | -.1589 | .0768 | -.3119, -.0058 |  |  |
|  | Subjective | 65 | -.2025 | .0284 | -.2592, -.1458 |  |  |
| 37 | Type of health outcome measurement - outlier removed | 73 |  |  |  | 0.6822 (1, 71) | .4116 |
|  | Objective | 10 | -.1461 | .0388 | -.2235, -.0687 |  |  |
|  | Subjective | 63 | -.1762 | .0175 | -.2111, -.1412 |  |  |
| 38 | Age category | 75 |  |  |  | 0.6975 (1, 73) | .4063 |
|  | Adults | 7 | -.1343 | .0837 | -.3011, .0325 |  |  |
|  | Older adults | 68 | -.2085 | .0299 | -.2682, -.1489 |  |  |
| 39 | Age category- outlier removed | 73 |  |  |  | 0.7754 (1, 71) | .3815 |
|  | Adults | 7 | -.1342 | .0494 | -.2327, -.0357 |  |  |
|  | Older adults | 66 | -.1807 | .0187 | -.2180, -.1434 |  |  |
| 40 | Socioeconomic Status (SES) | 39 |  |  |  | 0.7866 (2, 36) | .4630 |
|  | Low SES | 9 | -.3258 | .0904 | -.5092, -.1424 |  |  |
|  | Middle/high SES | 6 | -.2046 | .1041 | -.4157, .0066 |  |  |
|  | Mixed SES | 24 | -.1931 | .0575 | -.3097, -.0765 |  |  |
| 41 | SES- outlier removed | 37 |  |  |  | 0.7639 (2, 34) | .4737 |
|  | Low SES | 8 | -.2383 | .0519 | -.3439, -.1328 |  |  |
|  | Middle/high SES | 6 | -.1696 | .0405 | -.2519, -.0872 |  |  |
|  | Mixed SES | 23 | -.1638 | .0306 | -.2260, -.1017 |  |  |
| Use of services | | | | | | | |
| 42 | Publication year | 11 | -.0040 | .0054 | -.0162, .0081 | 0.5636 (1, 9) | .4720 |
| 43 | Continent | 11 |  |  |  | 2.8649 (2, 8) | .1153 |
|  | Asia | 3 | -.2576 | .1035 | -.4962, -.0190 |  |  |
|  | Europe | 4 | -.1462 | .0690 | -.3053, .0128 |  |  |
|  | North America | 4 | .0333 | .0773 | -.1450, .2116 |  |  |
| 44 | Gender | 11 | -.4071 | .3108 | -1.1103, .2960 | 1.7156 (1, 9) | .2227 |
| 45 | Loneliness scale | 11 |  |  |  | 1.4196 (1, 9) | .2639 |
|  | RTLS | 2 | -.2304 | .1284 | -.5210, .0601 |  |  |
|  | UCLA | 9 | -.0583 | .0662 | -.2079, .0914 |  |  |
| 46 | Type of health outcome measurement | 11 |  |  |  | 0.1771 (1, 9) | .6838 |
|  | Objective | 5 | -.0564 | .1006 | -.2839, .1711 |  |  |
|  | Subjective | 6 | -.1149 | .0958 | -.3316, .1019 |  |  |
| 47 | Age category | 11 |  |  |  | 0.0097 (1, 9) | .9236 |
|  | Adults | 5 | -.0795 | .1011 | -.3083, .1493 |  |  |
|  | Older adults | 6 | -.0933 | .0969 | -.3124, .1258 |  |  |
| 48 | Socioeconomic Status (SES) | 8 |  |  |  | 7.6088 (2, 5) | .0304 |
|  | Low SES | 2 | .0010 | .0649 | -.1660, .1679 |  |  |
|  | Middle/high SES | 1 | -.0055 | .0732 | -.1937, .1827 |  |  |
|  | Mixed SES | 5 | -.2415 | .0376 | -.3382, -.1448 |  |  |
| Sleep function | | | | | | | |
| 49 | Publication year | 50 | -.0035 | .0029 | -.0093, .0024 | 1.4158 (1, 48) | .2399 |
| 50 | Continent | 50 |  |  |  | 0.5570 (4, 45) | .6950 |
|  | Africa | 1 | -.1748 | .1480 | -.4729, .1234 |  |  |
|  | Asia | 15 | -.2319 | .0395 | -.3115, -.1523 |  |  |
|  | Europe | 4 | -.1878 | .0834 | -.3556, -.0199 |  |  |
|  | North America | 29 | -.1539 | .0412 | -.2370, -.0709 |  |  |
|  | Oceania | 1 | -.2986 | .1781 | -.6572, .0601 |  |  |
| 51 | Gender | 49 | .0150 | .1523 | -.2913, .3213 | 0.0097 (1, 47) | .9218 |
| 52 | Loneliness scale | 50 |  |  |  | 1.5482 (2, 47) | .2233 |
|  | Other | 2 | -.3820 | .1104 | -.6040, -.1599 |  |  |
|  | RTLS | 1 | -.1450 | .1461 | -.4389, .1489 |  |  |
|  | UCLA | 47 | -.1862 | .0265 | -.2395, -.1328 |  |  |
| 53 | Type of health outcome measurement | 50 |  |  |  | 0.1211 (1, 48) | .7294 |
|  | Objective | 3 | -.1715 | .0728 | -.3178, -.0252 |  |  |
|  | Subjective | 47 | -.1956 | .0258 | -.2474, -.1439 |  |  |
| 54 | Age category | 50 |  |  |  | 0.1924 (2, 47) | .8256 |
|  | Adults | 18 | -.2110 | .0396 | -.2906, -.1314 |  |  |
|  | Children and adolescents | 17 | -.1948 | .0504 | -.2961, -.0935 |  |  |
|  | Older adults | 15 | -.1712 | .0506 | -.2730, -.0695 |  |  |
| 55 | Socioeconomic Status (SES) | 11 |  |  |  | 0.8351 (2, 8) | .4684 |
|  | Low SES | 2 | .0051 | .1370 | -.3109, .3210 |  |  |
|  | Middle/high SES | 2 | -.1384 | .1294 | -.4369, .1601 |  |  |
|  | Mixed SES | 7 | -.1907 | .0659 | -.3426, -.0388 |  |  |
| Sensory acuity | | | | | | | |
| 57 | Publication year | 20 | .0030 | .0039 | -.0053, .0112 | 0.5741 (1, 18) | .4584 |
| 58 | Continent | 20 |  |  |  | 6.2044 (2, 17) | **.0095** |
|  | Asia | 4 | -.0311 | .0233 | -.0803, .0181 |  |  |
|  | Europe | 7 | -.1232 | .0320 | -.1906, -.0558 |  |  |
|  | North America | 9 | -.1333 | .0187 | -.1729, -.0938 |  |  |
| 59 | Gender | 20 | -.1646 | .2182 | -.6231, .2938 | 0.5693 (1, 18) | .4603 |
| 60 | Loneliness scale | 20 |  |  |  | 0.1649 (1, 18) | .6895 |
|  | RTLS | 4 | -.0999 | .0540 | -.2134, .0135 |  |  |
|  | UCLA | 16 | -.1239 | .0242 | -.1748 -.0731 |  |  |
| 61 | Type of health outcome measurement | 20 |  |  |  | 02.6918 (1, 18) | .1182 |
|  | Objective | 7 | -.0842 | .0296 | -.1463, -.0221 |  |  |
|  | Subjective | 13 | -.1363 | .0236 | -.1858, -.0867 |  |  |
| 62 | Socioeconomic Status (SES) | 10 |  |  |  | 0.0111 (1, 8) | .9188 |
|  | Middle/high SES | 6 | -.0898 | .0329 | -.1657, -.0138 |  |  |
|  | Mixed SES | 4 | -.0942 | .0389 | -.1839, -.0046 |  |  |

*Notes*. *p* < .01 is significant; k = number of effect sizes; *b* = regression coefficient; *SE* = standard error; CI = confidence interval; *F*(df1, df2) omnibus test; RTLS = Rasch-Type Loneliness Scale; UCLA = University of California Los Angeles Loneliness Scale. For the categorical variables, the provided regression coefficients reflect the mean effect sizes (Fisher’s *Z*) for each category.

**Figure S1**. Funnel plots of effect sizes by their standard errors


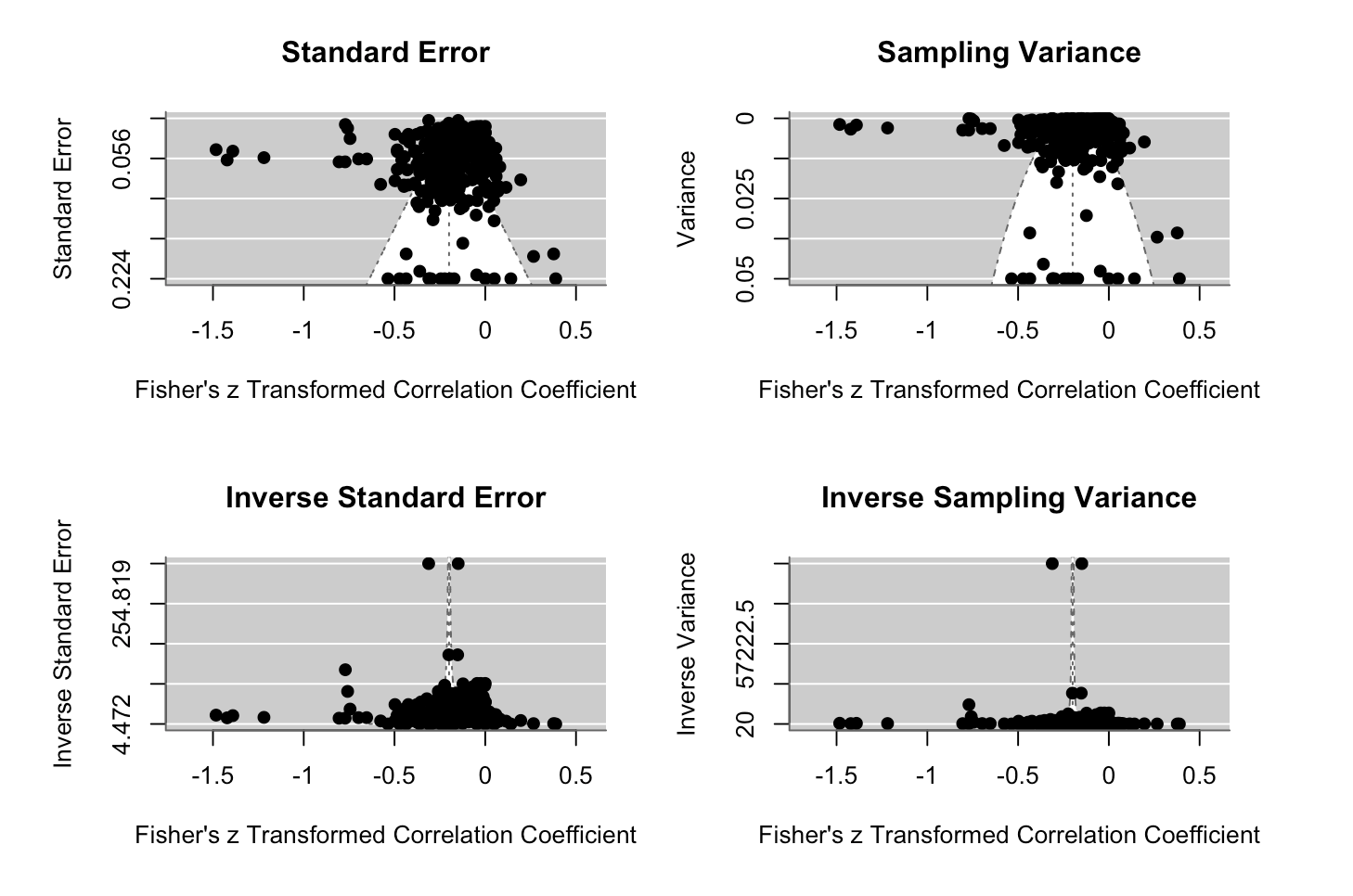


*Alt text*: Graphs showing the four funnel plots effect sizes by their standard errors, in the following order left to right, top to bottom: standard error, sampling variance, inverse standard error, and inverse sampling variance.

**Figure S2**. Funnel Plots of Effect Sizes by Their Standard Errors When Outliers Were Deleted


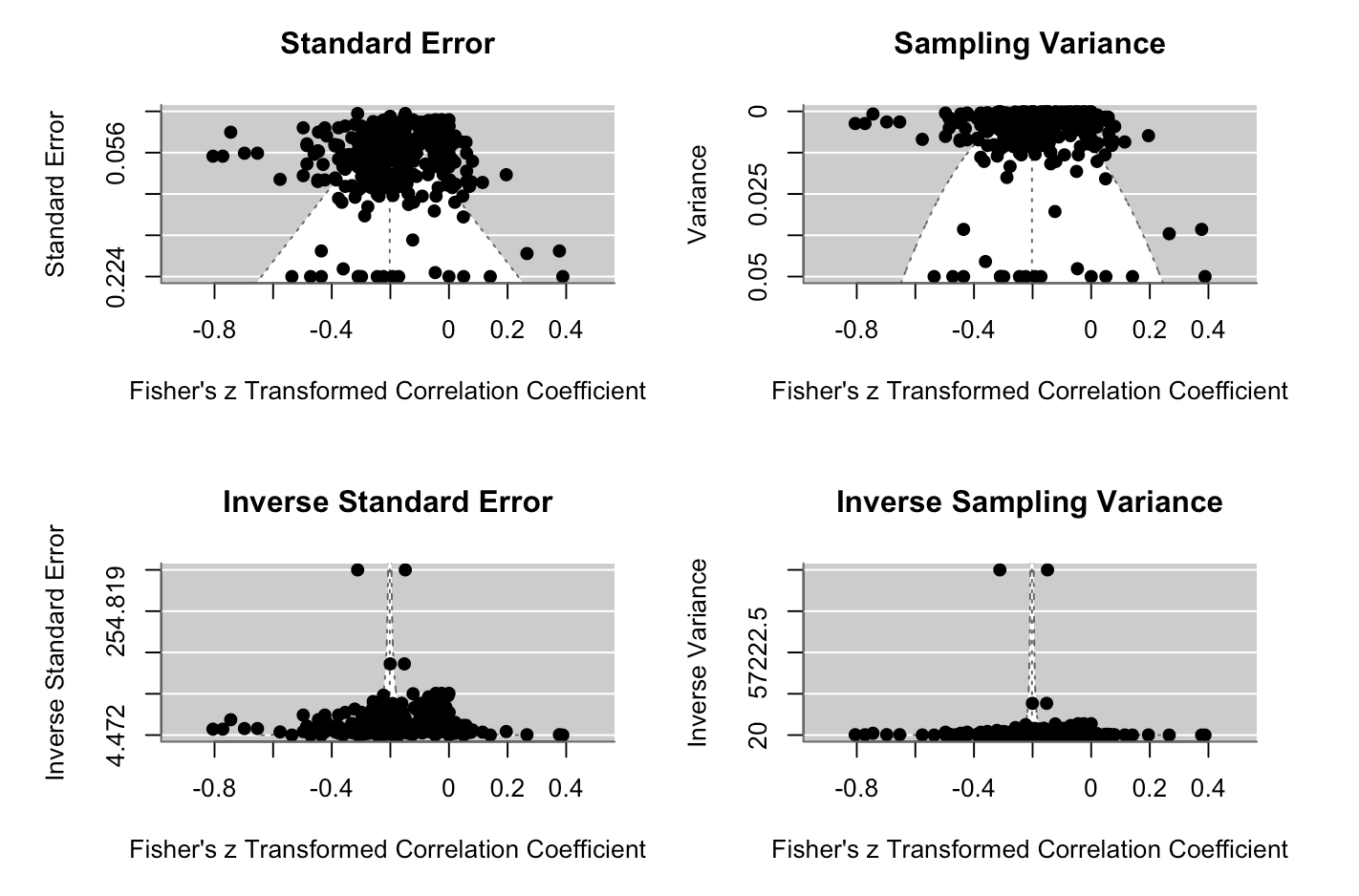


*Alt text*: Graphs showing the four funnel plots effect sizes by their standard errors, when outliers were deleted, in the following order left to right, top to bottom: standard error, sampling variance, inverse standard error, and inverse sampling variance
