# Supplementary material for: Impact of loneliness on health in healthy populations: A meta‐analysis
Source: Br J Health Psychol. 2025 Dec 7;31(1):e70040. doi: 10.1111/bjhp.70040 (PMC12683082; doi:10.1111/bjhp.70040)
Supplement: Supplementary file 2 — Data S2: [file BJHP-31-0-s002.docx]

**Supplementary Material S2**

**Variance Decomposition and Heterogeneity Across Levels in the Loneliness–Health Meta-Analysis**

**Including the Outliers**

***Global health***

The median sampling variance was 0.0031, accounting for 8.09% of the total variance. The Level 2 variance was 0.0188, LRT *χ²* (1) = 39.20, p < .0001, accounting for 48.55% of the total variance, indicating substantial within-sample variation. The Level 3 variance was 0.0000, LRT *χ²* (1) = 0.00, *p* = 1.00, accounting for 0.00% of the total variance, suggesting no meaningful heterogeneity at this level. The Level 4 variance was 0.0168, LRT *χ²* (1) = 3015.59, *p* < .0001, accounting for 43.35% of the total variance, indicating strong between-dataset heterogeneity.

***General health***

The median sampling variance was 0.0034, accounting for 7.30% of the total variance. The Level 2 variance was 0.0189, LRT *χ²* (1) = 26.39, *p* < .0001, accounting for 40.99% of the total variance, indicating substantial within-sample variation. The Level 3 variance was 0.0066, LRT *χ²* (1) = 1.04, *p* = .31, accounting for 14.42% of the total variance, but was not statistically significant. The Level 4 variance was 0.0172, LRT *χ²* (1) = 402.67, *p* < .0001, accounting for 37.29% of the total variance, indicating strong between-dataset heterogeneity.

***Physical health***

The median sampling variance was 0.0020, accounting for 5.15% of the total variance. The Level 2 variance was 0.0208, LRT *χ²* (1) = 3.65, *p* = .06, accounting for 53.03% of the total variance, indicating substantial within-sample variation. The Level 3 variance was 0.0000, LRT *χ²* (1) = 0.00, *p* = 1.00, accounting for 0.00% of the total variance, suggesting no meaningful heterogeneity at this level. The Level 4 variance was 0.0164, LRT *χ²* (1) = 162.96, *p* < .0001, accounting for 41.81% of the total variance, indicating strong between-dataset heterogeneity.

***Use of services***

The median sampling variance was 0.0010, accounting for 5.19% of the total variance. The Level 2 variance was 0.0151, LRT *χ²* (1) = 1.23, *p* = .27, accounting for 75.38% of the total variance, indicating substantial but non-significant within-sample variation. The Level 3 variance was 0.0000, LRT *χ²* (1) = 0.00, *p* = 1.00, accounting for 0.00% of the total variance, suggesting no meaningful heterogeneity at this level. The Level 4 variance was 0.0039, LRT *χ²* (1) = 1498.69, *p* < .0001, accounting for 19.43% of the total variance, indicating strong between-dataset heterogeneity.

***Sleep***

The median sampling variance was 0.0021, accounting for 9.58% of the total variance. The Level 2 variance was 0.0135, LRT *χ²* (1) = 2.56, *p* = .11, accounting for 60.34% of the total variance, indicating substantial but non-significant within-sample variation. The Level 3 variance was 0.0000, LRT *χ²* (1) = 0.00, *p* = 1.00, accounting for 0.00% of the total variance, suggesting no meaningful heterogeneity at this level. The Level 4 variance was 0.0067, LRT *χ²* (1) = 79.00, *p* < .0001, accounting for 30.09% of the total variance, indicating strong between-dataset heterogeneity.

***Sensory***

The median sampling variance was 0.0014, accounting for 25.27% of the total variance. The Level 2 variance was 0.0021, LRT *χ²* (1) = 2.17, *p* = .14, accounting for 38.12% of the total variance, indicating substantial but non-significant within-sample variation. The Level 3 variance was 0.0000, LRT *χ²* (1) = 0.00, *p* = 1.00, accounting for 0.00% of the total variance, suggesting no meaningful heterogeneity at this level. The Level 4 variance was 0.0020, LRT *χ²* (1) = 22.09, *p* < .0001, accounting for 36.61% of the total variance, indicating strong between-dataset heterogeneity.

**Excluding the Outliers**

***Global health***

The median sampling variance was 0.0032, accounting for 15.30% of the total variance. The Level 2 variance was 0.0096, LRT *χ²* (1) = 16.92, *p* < .0001, accounting for 45.71% of the total variance, indicating substantial within-sample variation. The Level 3 variance was 0.0000, LRT *χ²* (1) = 0.00, *p* = 1.00, accounting for 0.00% of the total variance, suggesting no meaningful heterogeneity at this level. The Level 4 variance was 0.0082, LRT *χ²* (1) = 2211.01, *p* < .0001, accounting for 38.99% of the total variance, indicating strong between-dataset heterogeneity.

***General health***

The median sampling variance was 0.0036, accounting for 14.52% of the total variance. The Level 2 variance was 0.0097, LRT *χ²* (1) = 8.78, *p* < .005, accounting for 38.63% of the total variance, indicating substantial within-sample variation. The Level 3 variance was 0.0000, LRT *χ²* (1) = 0.00, *p* = 1.00, accounting for 0.00% of the total variance, suggesting no meaningful heterogeneity at this level. The Level 4 variance was 0.0117, LRT *χ²* (1) = 392.19, *p* < .0001, accounting for 46.86% of the total variance, indicating strong between-dataset heterogeneity.

***Physical health***

The median sampling variance was 0.0020, accounting for 15.10% of the total variance. The Level 2 variance was 0.0092, LRT *χ²* (1) = 6.60, *p* = .01, accounting for 68.58% of the total variance, indicating substantial within-sample variation. The Level 3 variance was 0.0000, LRT *χ²* (1) = 0.00, *p* = 1.00, accounting for 0.00% of the total variance, suggesting no meaningful heterogeneity at this level. The Level 4 variance was 0.0022, LRT *χ²* (1) = 71.83, *p* < .0001, accounting for 16.31% of the total variance, indicating strong between-dataset heterogeneity.

***Use of services***

The median sampling variance was 0.0010, accounting for 5.19% of the total variance. The Level 2 variance was 0.0151, LRT *χ²* (1) = 1.23, *p* = .27, accounting for 75.38% of the total variance, indicating substantial but non-significant within-sample variation. The Level 3 variance was 0.0000, LRT *χ²* (1) = 0.00, *p* = 1.00, accounting for 0.00% of the total variance, suggesting no meaningful heterogeneity at this level. The Level 4 variance was 0.0039, LRT *χ²* (1) = 1498.69, *p* < .0001, accounting for 19.43% of the total variance, indicating strong between-dataset heterogeneity.

***Sleep***

The median sampling variance was 0.0021, accounting for 9.58% of the total variance. The Level 2 variance was 0.0135, LRT *χ²* (1) = 2.56, *p* = .11, accounting for 60.34% of the total variance, indicating substantial but non-significant within-sample variation. The Level 3 variance was 0.0000, LRT *χ²* (1) = 0.00, *p* = 1.00, accounting for 0.00% of the total variance, suggesting no meaningful heterogeneity at this level. The Level 4 variance was 0.0067, LRT *χ²* (1) = 79.00, *p* < .0001, accounting for 30.09% of the total variance, indicating strong between-dataset heterogeneity.

***Sensory***

The median sampling variance was 0.0014, accounting for 25.27% of the total variance. The Level 2 variance was 0.0021, LRT *χ²* (1) = 2.17, *p* = .14, accounting for 38.12% of the total variance, indicating substantial but non-significant within-sample variation. The Level 3 variance was 0.0000, LRT *χ²* (1) = 0.00, *p* = 1.00, accounting for 0.00% of the total variance, suggesting no meaningful heterogeneity at this level. The Level 4 variance was 0.0020, LRT *χ²* (1) = 22.09, *p* < .0001, accounting for 36.61% of the total variance, indicating strong between-dataset heterogeneity.
