# Supplementary material for: Impact of loneliness on health in healthy populations: A meta‐analysis
Source: Br J Health Psychol. 2025 Dec 7;31(1):e70040. doi: 10.1111/bjhp.70040 (PMC12683082; doi:10.1111/bjhp.70040)
Supplement: Supplementary file 3 — Data S3: [file BJHP-31-0-s003.docx]

**Supplementary References**

1. Adams KB, Sanders S, Auth EA. Loneliness and depression in independent living retirement communities: risk and resilience factors. *Aging & Mental Health* 2004;8(6):475-85.
2. Auslander GK, Soffer M, Auslander BA. The supportive community: help seeking and service use among elderly people in Jerusalem. *Social Work Research* 2003;27(4):209-21.
3. Baum SK. Loneliness in elderly persons: a preliminary study. *Psychological Reports* 1982;50(3_suppl):1317-8.
4. Bookwala J. Marital quality as a moderator of the effects of poor vision on quality of life among older adults. *Journals of Gerontology Series B: Psychological Sciences and Social Sciences* 2011;66(5):605-16.
5. Bookwala J, Lawson B. Poor vision, functioning, and depressive symptoms: a test of the activity restriction model. *The Gerontologist* 2011;51(6):798-808.
6. Chalise HN, Saito T, Kai I. Correlates of loneliness among older Newar adults in Nepal. *Nihon Koshu Eisei Zasshi (Japanese Journal of Public Health)* 2007;54(7):427-33.
7. Downs VC, Javidi M, Nussbaum JF. A comparative analysis of the relationship between communication apprehension and loneliness for elderly nursing home and non‐nursing home residents. *Western Journal of Speech Communication* 1988;52(4):308-20.
8. Durak M, Senol-Durak E. Psychometric qualities of the UCLA Loneliness Scale-version 3 as applied in a Turkish culture. *Educational Gerontology* 2010;36(10-11):988-1007. <https://doi.org/10.1080/03601271003756628>
9. Ebrecht M, Hextall J, Kirtley LG, Taylor A, Dyson M, Weinman J. Perceived stress and cortisol levels predict speed of wound healing in healthy male adults. *Psychoneuroendocrinology* 2004;29(6):798-809. <https://doi.org/10.1016/s0306-4530(03)00144-6>
10. Fauth EB, Zarit SH, Malmberg B. Mediating relationships within the disablement process model: a cross-sectional study of the oldest-old. *European Journal of Ageing* 2008;5:161-79.
11. Fernández-Alonso AM, Trabalón-Pastor M, Vara C, Chedraui P, Pérez-López FR, MenopAuse RIsk Assessment (MARIA) Research Group. Life satisfaction, loneliness and related factors during female midlife. *Maturitas* 2012;72(1):88-92. <https://doi.org/10.1016/j.maturitas.2012.02.001>
12. Finkenauer C, Engels RC, Meeus W. Keeping secrets from parents: advantages and disadvantages of secrecy in adolescence. *Journal of Youth and Adolescence* 2002;31:123-36.
13. Freund AM, Baltes PB. Selection, optimization, and compensation as strategies of life management: correlations with subjective indicators of successful aging. *Psychology and Aging* 1998;13(4):531.
14. Gerstorf D, Smith J, Baltes PB. A systemic-wholistic approach to differential aging: longitudinal findings from the Berlin Aging Study. *Psychology and Aging* 2006;21(4):645.
15. Hansson RO, Jones WH, Carpenter BN, Remondet JH. Loneliness and adjustment to old age. *The International Journal of Aging and Human Development* 1987;24(1):41-53.
16. Hawkley LC, Preacher KJ, Cacioppo JT. Loneliness impairs daytime functioning but not sleep duration. *Health Psychology* 2010;29(2):124.
17. Hays RD, DiMatteo MR. A short-form measure of loneliness. *Journal of Personality Assessment* 1987;51(1):69-81.
18. Jaremka LM, Fagundes CP, Glaser R, Bennett JM, Malarkey WB, Kiecolt-Glaser JK. Loneliness predicts pain, depression, and fatigue: understanding the role of immune dysregulation. *Psychoneuroendocrinology* 2013;38(8):1310-7.
19. Kahn JH, Hessling RM, Russell DW. Social support, health, and well-being among the elderly: what is the role of negative affectivity?. *Personality and individual Differences* 2003;35(1):5-17.
20. Kidd S, Shahar G. Resilience in homeless youth: the key role of self-esteem. *American Journal of Orthopsychiatry* 2008;78(2):163.
21. Kuwert P, Knaevelsrud C, Pietrzak RH. Loneliness among older veterans in the United States: results from the National Health and Resilience in Veterans Study. *The American Journal of Geriatric Psychiatry* 2014;22(6):564-9.
22. Lawler‐Row KA, Hyatt‐Edwards LA, Wuensch KL, Karremans JC. Forgiveness and health: the role of attachment. *Personal Relationships* 2011;18(2):170-83.
23. Light AE, Visser PS. The ins and outs of the self: contrasting role exits and role entries as predictors of self-concept clarity. *Self and Identity* 2013;12(3):291-306.
24. Liu LJ, Guo Q. Loneliness and health-related quality of life for the empty nest elderly in the rural area of a mountainous county in China. *Quality of Life Research* 2007;16:1275-80.
25. Long MV, Martin P. Personality, relationship closeness, and loneliness of oldest old adults and their children. *The Journals of Gerontology Series B: Psychological Sciences and Social Sciences* 2000;55(5):P311-9.
26. Lutgendorf SK, Russell D, Ullrich P, Harris TB, Wallace R. Religious participation, interleukin-6, and mortality in older adults. *Health Psychology* 2004;23(5):465.
27. Mahon NE. Loneliness and sleep during adolescence. *Perceptual and Motor Skills* 1994;78(1):227-31.
28. Mahon NE, Yarcheski A, Yarcheski TJ. Loneliness and health-related variables in early adolescents: an extension. *Psychological Reports*  2003;93(1):233-4.
29. Mahon NE, Yarcheski TJ, Yarcheski A. Loneliness and health-related variables in young adults. *Perceptual and Motor Skills* 1997;85(3):800-2.
30. Margrett JA, Daugherty K, Martin P. et al. Affect and loneliness among centenarians and the oldest old: the role of individual and social resources. *Aging & Mental Health* 2011;15(3):385-96.
31. Matthews-Ewald MR, Zullig KJ. Evaluating the performance of a short loneliness scale among college students. *Journal of College Student Development*  2013;54(1):105-9.
32. McConnell AR, Brown CM, Shoda TM, Stayton LE, Martin CE. Friends with benefits: on the positive consequences of pet ownership. *Journal of Personality and Social Psychology* 2011;101(6):1239.
33. Nausheen B, Gidron Y, Gregg A, Tissarchondou HS, Peveler R. Loneliness, social support and cardiovascular reactivity to laboratory stress. *Stress* 2007;10(1):37-44.
34. Pereira MG, Taysi E, Orcan F, Fincham F. Attachment, infidelity, and loneliness in college students involved in a romantic relationship: the role of relationship satisfaction, morbidity, and prayer for partner. *Contemporary Family Therapy* 2013;36:333-50.
35. Plouffe L, Jomphe-Hill A. Distressed and lonely: health and social determinants among residents in seniors’ housing. *Journal of Clinical Geropsychology* 1996;2:51-60.
36. Poulin J, Deng R, Ingersoll TS, Witt H, Swain M. Perceived family and friend support and the psychological well-being of American and Chinese elderly persons. *Journal of Cross-Cultural Gerontology* 2012;27:305-17.
37. Reichl C, Schneider JF, Spinath FM. Relation of self-talk frequency to loneliness, need to belong, and health in German adults. *Personality and Individual Differences*  2013;54(2):241-5.
38. Reis HT, Wheeler L, Kernis MH, Spiegel N, Nezlek J. On specificity in the impact of social participation on physical and psychological health. *Journal of Personality and Social Psychology* 1985;48(2):456.
39. Scott SB, Jackson BR, Bergeman CS. What contributes to perceived stress in later life? A recursive partitioning approach. *Psychology and Aging* 2011;26(4):830.
40. Scott SB, Whitehead BR, Bergeman CS, Pitzer L. Combinations of stressors in midlife: examining role and domain stressors using regression trees and random forests. *Journals of Gerontology Series B: Psychological Sciences and Social Sciences* 2013;68(3):464-75.
41. Segrin C, Domschke T. Social support, loneliness, recuperative processes, and their direct and indirect effects on health. *Health Communication* 2011;26(3):221-32.
42. Segrin C, Passalacqua SA. Functions of loneliness, social support, health behaviors, and stress in association with poor health. *Health Communication* 2010;25(4):312-22.
43. Smith SS, Kozak N, Sullivan KA. An investigation of the relationship between subjective sleep quality, loneliness and mood in an Australian sample: can daily routine explain the links?. *International Journal of Social Psychiatry* 2012;58(2):166-71.
44. Springer RM, Weaver AJ, Linderblatt RC. et al. Spirituality, depression, and loneliness among Jewish seniors residing in New York City. Journal of Pastoral Care & Counseling 2003;57(3):305-18.
45. Staight PR, Harvey SM. Caregiver burden: a comparison between elderly women as primary and secondary caregivers for their spouses. *Journal of Gerontological Social Work* 1990;15(1-2):89-104.
46. Sun F, Waldron V, Gitelson R, Ho CH. The effects of loss of loved ones on life satisfaction among residents in a southwest retirement community: the mediating roles of social connectedness. *Research on Aging*  2012;34(2):222-45.
47. Utz RL, Lund DA, Caserta MS, Devries B. Perceived self-competency among the recently bereaved. *Journal of Social Work in End-of-Life & Palliative Care* 2011;7(2-3):173-94.
48. Walker D, Beauchene RE. The relationship of loneliness, social isolation, and physical health to dietary adequacy of independently living elderly. *Journal of the American Dietetic Association* 1991;91(3):300–4.
49. Wen M, Hawkley LC, Cacioppo JT. Objective and perceived neighborhood environment, individual SES and psychosocial factors, and self-rated health: An analysis of older adults in Cook County, Illinois. *Social Science & Medicine* 2006;63(10):2575-90.
50. Xu Z, Su H, Zou Y, Chen J, Wu J, Chang W. Self‐rated health of Chinese adolescents: distribution and its associated factors. *Scandinavian Journal of Caring Sciences* 2011;25(4):780-6.
51. Xu Z, Su H, Zou Y, Chen J, Wu J, Chang W. Sleep quality of Chinese adolescents: distribution and its associated factors. *Journal of Paediatrics and Child Health* 2012;48(2):138-45.
52. Alpass FM, Neville S. Loneliness, health and depression in older males. *Aging & Mental Health* 2003;7(3):212-6.
53. Habersaat SA, Geiger AM, Abdellaoui S, Wolf JM. Health in police officers: role of risk factor clusters and police divisions. *Social Science & Medicine* 2015;143:213-22.
54. Tse MMY, Wan VTC, Vong SKS. Health-related profile and quality of life among nursing home residents: does pain matter? *Pain Management Nursing* 2013;14(4):e173–84.
55. Wagner J, Hoppmann C, Ram N, Gerstorf D. Self-esteem is relatively stable late in life: the role of resources in the health, self-regulation, and social domains. *Developmental Psychology*  2015;51(1):136.
56. Nishina A, Juvonen J, Witkow MR. Sticks and stones may break my bones, but names will make me feel sick: the psychosocial, somatic, and scholastic consequences of peer harassment. *Journal of Clinical Child and Adolescent Psychology* 2005;34(1):37-48.
57. Carcedo RJ, Perlman D, Orgaz MB, López F, Fernández-Rouco N, Faldowski RA. Heterosexual romantic relationships inside of prison: partner status as predictor of loneliness, sexual satisfaction, and quality of life. *International Journal of Offender Therapy and Comparative Criminology* 2011;55(6):898-924.
58. Bielderman A, van der Schans CP, van Lieshout MR. et al. Multidimensional structure of the Groningen Frailty Indicator in community-dwelling older people. *BMC Geriatrics*  2013;13:1-9.
59. Burke KE, Schnittger R, O’Dea B, Buckley V, Wherton JP, Lawlor BA. Factors associated with perceived health in older adult Irish population. *Aging & Mental Health* 2012;16(3):288-95.
60. De Donder L, De Witte N, Dury S, Buffel T, Verté D. Individual risk factors of feelings of unsafety in later life. *European Journal of Ageing* 2012;9:233-42.
61. De Jong Gierveld J, Van Tilburg T. A 6-item scale for overall, emotional, and social loneliness: confirmatory tests on survey data. *Research on Aging* 2006;28(5):582-98.
62. Eisses AM, Kluiter H, Jongenelis K, Pot AM, Beekman AT, Ormel J. Risk indicators of depression in residential homes. *International Journal of Geriatric Psychiatry* 2004;19(7):634-40.
63. Iecovich E. Psychometric properties of the Hebrew version of the de Jong Gierveld Loneliness Scale. *Educational Gerontology* 2013;39(1):12-27.
64. Newall NE, Chipperfield JG, Clifton RA, Perry RP, Swift AU, Ruthig JC. Causal beliefs, social participation, and loneliness among older adults: a longitudinal study. *Journal of Social and Personal Relationships* 2009;26(2-3):273-90.
65. Sadler EA, Braam AW, Broese van Groenou MI, Deeg DJ, Van der Geest S. Cosmic transcendence, loneliness, and exchange of emotional support with adult children: a study among older parents in The Netherlands. *European Journal of Ageing*  2006;3:146-54.
66. Schnittger RI, Wherton J, Prendergast D, Lawlor BA. Risk factors and mediating pathways of loneliness and social support in community-dwelling older adults. *Aging & Mental Health* 2012;16(3):335-46.
67. Steverink N, Lindenberg S. Do good self-managers have less physical and social resource deficits and more well-being in later life?. *European Journal of Ageing* 2008;5:181-90.
68. Van der Hal-van Raalte E, Van IJzendoorn MH, Bakermans-Kranenburg MJ. Quality of care after early childhood trauma and well-being in later life: child Holocaust survivors reaching old age. *American Journal of Orthopsychiatry* 2007;77(4):514–22.
69. Warner LM, Schüz B, Wurm S, Ziegelmann JP, Tesch-Römer C. Giving and taking-differential effects of providing, receiving and anticipating emotional support on quality of life in adults with multiple illnesses. *Journal of Health Psychology* 2010;15(5):660-70.
70. De Jong Gierveld J, Van Tilburg T. Living arrangements of older adults in the Netherlands and Italy: coresidence values and behaviour and their consequences for loneliness. *Journal of Cross-Cultural Gerontology* 1999;14:1-24.
71. Dahlberg L, McKee KJ. Correlates of social and emotional loneliness in older people: evidence from an English community study. *Aging & Mental Health* 2014;18(4):504-14.
72. Russell DW. UCLA Loneliness Scale (Version 3): reliability, validity, and factor structure. *Journal of Personality Assessment* 1996;66(1):20-40.
73. Steed L, Boldy D, Grenade L, Iredell H. The demographics of loneliness among older people in Perth, Western Australia. *Australasian Journal on Ageing* 2007;26(2):81-6.
74. Becker SP. External validity of children's self-reported sleep functioning: associations with academic, social, and behavioral adjustment. *Sleep Medicine* 2014;15(9):1094-100.
75. Chen Y, Hicks A, While AE. Validity and reliability of the modified Chinese version of the Older People's Quality of Life Questionnaire (OPQOL) in older people living alone in China. *International Journal of Older People Nursing*  2014;9(4):306-16.
76. Gerich J. Effects of social networks on health from a stress theoretical perspective. *Social Indicators Research*  2014;118(1):349-64.
77. Schumaker JF, Krejci RC, Small L, Sargent RG. Experience of loneliness by obese individuals. *Psychological Reports* 1985;57(3_suppl):1147-54.
78. Hartung FM, Renner B. The need to belong and the relationship between loneliness and health. Zeitschrift für Gesundheitspsychologie 2014;22(4):194-201.
79. Balter LJ, Raymond JE, Aldred S. et al. Loneliness in healthy young adults predicts inflammatory responsiveness to a mild immune challenge in vivo. *Brain, Behavior, and Immunity* 2019;82:298-301.
80. Bowen CE, Luy M. Community social characteristics and health at older ages: evidence from 156 religious communities. *The Journals of Gerontology: Series B* 2018;73(8):1429-38.
81. Cimarolli VR, Jopp DS, Boerner K, Minahan J. Depressive symptoms in the oldest-old: the role of sensory impairments. *Archives of Gerontology and Geriatrics* 2018;78:249-54.
82. Corona K, Campos B, Chen C. Familism is associated with psychological well-being and physical health: main effects and stress-buffering effects. *Hispanic Journal of Behavioral Sciences*  2017;39(1):46-65.
83. Corona K, Senft N, Campos B, Chen C, Shiota M, Chentsova-Dutton YE. Ethnic variation in gratitude and well-being. *Emotion* 2020;20(3):518.
84. Gonyea JG, Curley A, Melekis K, Levine N, Lee Y. Loneliness and depression among older adults in urban subsidized housing. *Journal of Aging and Health* 2016;30(3):458-74.
85. Jung FU, Luck-Sikorski C. Overweight and lonely? A representative study on loneliness in obese people and its determinants. *Obesity Facts* 2019;12(4):440-7.
86. Lee CY, Goldstein SE, Dik BJ, Rodas JM. Sources of social support and gender in perceived stress and individual adjustment among Latina/o college-attending emerging adults. *Cultural Diversity and Ethnic Minority Psychology* 2020;26(1):134.
87. Lin X, Bryant C, Boldero J, Dow B. Psychological well-being of older Chinese immigrants living in Australia: a comparison with older Caucasians. *International Psychogeriatrics* 2016;28(10):1671-9.
88. Mund M, Neyer FJ. The winding paths of the lonesome cowboy: evidence for mutual influences between personality, subjective health, and loneliness. *Journal of Personality* 2016;84(5):646-57.
89. Schutter N, Holwerda TJ, Stek ML, Dekker JJ, Rhebergen D, Comijs HC. Loneliness in older adults is associated with diminished cortisol output. *Journal of Psychosomatic Research* 2017;95:19-25.
90. Steel JL, Cheng H, Pathak R. et al. Psychosocial and behavioral pathways of metabolic syndrome in cancer caregivers. *Psycho‐Oncology* 2019;28(8):1735-42.
91. Tan SS, Fierloos IN, Zhang X. et al. The association between loneliness and health related quality of life (HR-QoL) among community-dwelling older citizens. *International Journal of Environmental Research and Public Health* 2020;17(2):600.
92. Tully MA, McMullan II, Blackburn NE. et al. Is sedentary behavior or physical activity associated with loneliness in older adults? Results of the European-wide SITLESS study. *Journal of Aging and Physical Activity* 2019;28(4):549-55.
93. Yang X, Fan C, Liu Q, Chu X, Song Y, Zhou Z. Parenting styles and children’s sleep quality: examining the mediating roles of mindfulness and loneliness. *Children and Youth Services Review*  2020;114:104921.
94. Burns A, Leavey G, Ward M, O’Sullivan R. The impact of loneliness on healthcare use in older people: evidence from a nationally representative cohort. *Journal of Public Health* 2022:1-0.
95. Hajek A, König HH. Do lonely and socially isolated individuals think they die earlier? The link between loneliness, social isolation and expectations of longevity based on a nationally representative sample. *Psychogeriatrics* 2021;21(4):571-6.
96. Ho TT, Huynh SV, Tran-Chi VL. Impact of problematic Facebook use, loneliness, and poor sleep quality on mental health. *International Journal of Advanced and Applied Sciences* 2021;8(9):112-8.
97. Kino S, Stickley A, Arakawa Y, Saito M, Saito T, Kondo N. Social isolation, loneliness, and their correlates in older Japanese adults. *Psychogeriatrics*  2023;23(3):475-86.
98. Hajek A, König HH. Prevalence and correlates of loneliness, perceived and objective social isolation during the COVID-19 pandemic. Evidence from a representative survey in Germany. *Social Psychiatry and Psychiatric Epidemiology* 2022;57(10):1969-78.
99. Hisata Y, Sugioka T, Yasaka A. et al. Negative association between loneliness and healthy state among rural residents in Japan: a cross-sectional single region population-based survey. *Journal of Rural Medicine* 2023;18(2):70-8.
100. Kim YB, Lee SH. Gender differences in correlates of loneliness among community-dwelling older Koreans. *International Journal of Environmental Research and Public Health* 2022;19(12):7334.
101. Gyasi RM, Asamoah E, Gyasi-Boadu N, Zornu O, Asiki G, Phillips DR. Food insecurity and sleep quality among older adults: Findings from a population-based study in Ghana. *Maturitas* 2022 Mar;157:27-33.
102. Gyasi RM, Peprah P, Abass K. et al. Loneliness and physical function impairment: perceived health status as an effect modifier in community-dwelling older adults in Ghana. *Preventive Medicine Reports* 2022;26:101721.
103. Hussein SZ, Ismail AH, Abu Bakar SH. Loneliness and health outcomes among Malaysian older adults. *Makara Journal of Health Research* 2021;25(2):3.
104. Lu M, Bronskill SE, Strauss R. et al. Factors associated with loneliness in immigrant and Canadian-born older adults in Ontario, Canada: a population-based study. *BMC Geriatrics* 2023;23(1):380.
105. Ozcan H, Savci H, Canik M, Ayan M, Bas CF. Comparison of the relationship between menopausal symptoms and loneliness and anger. *Perspectives in Psychiatric Care* 2022;58(4):1900-6.
106. Nguyen TT, Zhang X, Wu TC. et al. Association of loneliness and wisdom with gut microbial diversity and composition: an exploratory study. *Frontiers in Psychiatry* 2021;12:648475.
107. Meisters R, Westra D, Putrik P, Bosma H, Ruwaard D, Jansen M. Does loneliness have a cost? A population-wide study of the association between loneliness and healthcare expenditure. *International Journal of Public Health* 2021;66:581286.
108. Mead MP, Vargas EA, Knutson KL. Racial disparities in sleep: potential mediation by discrimination and psychological distress. *Journal of Racial and Ethnic Health Disparities* 2023;10(2):573-80.
109. Masaeli N, Farhadi H. Internet addiction and depression in İran: investigating the mediating roles of loneliness and disordered sleep and moderating role of gender. *International Journal for the Advancement of Counselling* 2021;43(4):407-23.
110. Lutzman M, Sommerfeld E, Ben-David S. Loneliness and social integration as mediators between physical pain and suicidal ideation among elderly men. *International Psychogeriatrics* 2021;33(5):453-9.
111. Luo X, Hu C. Loneliness and sleep disturbance among first‐year college students: the sequential mediating effect of attachment anxiety and mobile social media dependence. *Psychology in the Schools* 2022;59(9):1776-89.
112. Loughrey DG, Mihelj E, Lawlor BA. Age-related hearing loss associated with altered response efficiency and variability on a visual sustained attention task. *Aging, Neuropsychology, and Cognition* 2021;28(1):1-25.
113. Littlejohn J, Venneri A, Marsden A, Plack CJ. Self-reported hearing difficulties are associated with loneliness, depression and cognitive dysfunction during the COVID-19 pandemic. *International Journal of Audiology* 2022;61(2):97-101.
114. Lin C, Glynn NW, Gmelin T. et al. Validation of the traditional Chinese version of the Pittsburgh fatigability scale for older adults. *Clinical Gerontologist* 2022;45(3):606-18.
115. Kuang K, Huisingh‐Scheetz M, Miller MJ, Waite L, Kotwal AA. The association of gait speed and self‐reported difficulty walking with social isolation: a nationally‐representative study. *Journal of the American Geriatrics Society* 2023;71(8):2549-56.
116. Krobisch V, Gebert P, Gül K, Schenk L. Women bear a burden: gender differences in health of older migrants from Turkey. *European Journal of Ageing* 2021;18(4):467-78.
117. Killgore WD, Grandner MA, Tubbs AS. et al. Sleep loss suicidal ideation: the role of trait extraversion. *Frontiers in Behavioral Neuroscience* 2022;16:886836.
118. Jopling E, Rnic K, Tracy A, LeMoult J. Impact of loneliness on diurnal cortisol in youth. *Psychoneuroendocrinology*  2021;132:105345.
119. Johar H, Atasoy S, Bidlingmaier M, Henningsen P, Ladwig KH. Married but lonely. Impact of poor marital quality on diurnal cortisol patterns in older people: findings from the cross-sectional KORA-Age study. *Stress* 2021;24(1):36-43.
120. Jiang F, Zhang J, Qin W, Ding G, Xu L. Hearing impairment and loneliness in older adults in Shandong, China: the modifying effect of living arrangement. *Aging Clinical and Experimental Research* 2021;33:1015-21.
121. Jiang F, Kuper H, Zhou C, Qin W, Xu L. Relationship between hearing loss and depression symptoms among older adults in China: the mediating role of social isolation and loneliness. *International Journal of Geriatric Psychiatry* 2022;37(6).
122. Huang AR, Deal JA, Rebok GW, Pinto JM, Waite L, Lin FR. Hearing impairment and loneliness in older adults in the United States*. Journal of Applied Gerontology*  2021;40(10):1366-71.
123. Hofman A, Overberg RI, Schoenmakers EC, Adriaanse MC. Social and emotional loneliness in a large sample of Dutch adults aged 19-65: associations with risk factors. *Psychiatry Research*  2022;313:114602.
124. Hawkley LC, Buecker S, Kaiser T, Luhmann M. Loneliness from young adulthood to old age: explaining age differences in loneliness*. International Journal of Behavioral Development* 2022;46(1):39-49.
125. Hafstad GS, Sætren SS, Wentzel-Larsen T, Augusti EM. Changes in adolescent mental and somatic health complaints throughout the COVID-19 pandemic: a three-wave prospective longitudinal study. *Journal of Adolescent Health* 2022;71(4):406-13.
126. Abu Elheja R, Palgi Y, Feldman R, Zagoory-Sharon O, Keisari S, Shamay-Tsoory SG. The role of oxytocin in regulating loneliness in old age. *Psychoneuroendocrinology* 2021;133:105413.
127. Albert I. Perceived loneliness and the role of cultural and intergenerational belonging: the case of Portuguese first-generation immigrants in Luxembourg. *European Journal of Ageing* 2021;18(3):299-310.
128. Baumbach L, König HH, Hajek A. Associations between changes in physical activity and perceived social exclusion and loneliness within middle-aged adults–longitudinal evidence from the German ageing survey. *BMC Public Health* 2023;23(1):274.
129. Benson JA, McSorley VE, Hawkley LC, Lauderdale DS. Associations of loneliness and social isolation with actigraph and self-reported sleep quality in a national sample of older adults. *Sleep* 2021;44(1):zsaa140.
130. Bergman YS, Shrira A, Palgi Y, Shmotkin D. The moderating role of the hostile-world scenario in the connections between COVID-19 worries, loneliness, and anxiety. *Frontiers in Psychology* 2021;12:645655.
131. Chang H, Huang Y, Liu X. Loneliness status and related factors among the Hakka elderly in Fujian, China: based on the health ecological model. *Journal of Environmental and Public Health* 2022;2022(1):2633297.
132. Chong EY, Lim AH, Mah FC, Yeo LH, Ng ST, Yi H. Assessing the psychosocial dimensions of frailty among older adults in Singapore: a community-based cross-sectional study. *BMJ Open* 2022;12(2):e047586.
133. Crespo-Sanmiguel I, Zapater-Fajarí M, Garrido-Chaves R, Hidalgo V, Salvador A. Loneliness and health indicators in middle-aged and older females and males. *Frontiers in Behavioral Neuroscience*  2022;16:809733.
134. Czaja SJ, Moxley JH, Rogers WA. Social support, isolation, loneliness, and health among older adults in the PRISM randomized controlled trial. *Frontiers in Psychology* 2021;12:728658.
135. Das R, Hasan MR, Daria S, Islam MR. Impact of COVID-19 pandemic on mental health among general Bangladeshi population: a cross-sectional study. *BMJ Open* 2021;11(4):e045727.
136. Encarnação S, Vaz P, Fortunato Á, Forte P, Vaz C, Monteiro AM. Aerobic fitness as an important moderator risk factor for loneliness in physically trained older people: an explanatory case study using machine learning. *Life* 2023;13(6):1374.
137. Grossman ES, Hoffman YS, Palgi Y, Shrira A. COVID-19 related loneliness and sleep problems in older adults: worries and resilience as potential moderators. *Personality and Individual Differences* 2021;168:110371.
138. Gu S, He Z, Sun L. et al. Effects of Coronavirus-19 induced loneliness on mental health: sleep quality and intolerance for uncertainty as mediators. *Frontiers in Psychiatry*  2021;12:738003.
139. Xiao S, Shi L, Xue Y. et al. The relationship between activities of daily living and psychological distress among Chinese older adults: a serial multiple mediation model. *Journal of Affective Disorders* 2022;300:462-8.
140. Zilioli S, Jiang Y. Endocrine and immunomodulatory effects of social isolation and loneliness across adulthood. *Psychoneuroendocrinology* 2021;128:105194.
141. Yu K, Wu S, Jang Y. et al. Longitudinal assessment of the relationships between geriatric conditions and loneliness. *Journal of the American Medical Directors Association* 2021;22(5):1107-13.
142. Rufarakh A, Majeed S, Jahangir A, Khan MJ, Farooq Z, Mohammad A. Effect of loneliness and sleep disturbances on mental health problems among young adults during COVID-19 pandemic: moderating role of resilience. *Rawal Medical Journal* 2021;46(4):776-.
143. Van Bogart K, Engeland CG, Sliwinski MJ. et al. The association between loneliness and inflammation: findings from an older adult sample. *Frontiers in Behavioral Neuroscience* 2022;15:801746.
144. Yuan K, Luo Y, Sun J, Chang H, Hu H, Zhao B. Depression and cognition mediate the effect of self-perceptions of aging over frailty among older adults living in the community in China. *Frontiers in Psychology* 2022;13:830667.
145. Stout ME, Keirns BH, Hawkins MA. Hypocortisolemic reactivity to acute social stress among lonely young women. *Social Neuroscience* 2023;18(3):171-82.
146. Yang H, Tng GY, Ng WQ, Yang S. Loneliness, sense of control, and risk of dementia in healthy older adults: a moderated mediation analysis. *Clinical Gerontologist* 2021;44(4):392-405.
147. Pataka A, Kotoulas S, Tzinas A. et al. Sleep disorders and mental stress of healthcare workers during the two first waves of COVID-19 pandemic: separate analysis for primary care. *Healthcare* 2022;10(8):1395.
148. Perez LG, Siconolfi D, Troxel WM. et al. Loneliness and multiple health domains: associations among emerging adults. *Journal of Behavioral Medicine*  2022;45(2):260-71.
149. Savci C, Akinci AC, Usenmez SY, Keles F. The effects of fear of COVID-19, loneliness, and resilience on the quality of life in older adults living in a nursing home. *Geriatric Nursing* 2021;42(6):1422-8.
150. Tümer A, Dönmez S, Gümüşsoy S, Balkaya NA. The relationship among aging in place, loneliness, and life satisfaction in the elderly in Turkey. *Perspectives in Psychiatric Care* 2022;58(2):822-9.
151. Takeda T, Yoshimi K, Kai S, Inoue F. Association between loneliness, premenstrual symptoms, and other factors during the COVID-19 pandemic: a cross-sectional study with Japanese high school students. *International Journal of Women's Health* 2023:655-64.
152. Ten Kate R, Bilecen B, Steverink N. The role of parent‐child relationships and filial expectations in loneliness among older Turkish migrants. *Social Inclusion* 2021;9(4):291-303.
153. Tian H, Wang Y. Mobile phone addiction and sleep quality among older people: the mediating roles of depression and loneliness. *Behavioral Sciences* 2023;13(2):153.
154. Turner-Cobb JM, Arden-Close E, Portch E, Wignall L. Men and women as differential social barometers: gender effects of perceived friend support on the neuroticism-loneliness-well-being relationship in a younger adult population. *International Journal of Environmental Research and Public Health* 2022;19(13):7986.
155. Zwilling M. The impact of nomophobia, stress, and loneliness on smartphone addiction among young adults during and after the COVID-19 pandemic: an Israeli case analysis. *Sustainability* 2022;14(6):3229.
156. Rumas R, Shamblaw AL, Jagtap S, Best MW. Predictors and consequences of loneliness during the COVID-19 pandemic. *Psychiatry Research* 2021;300:113934.
157. Zawadzki MJ, Gavrilova L. All the lonely people: comparing the effects of loneliness as a social stressor to non-lonely stress on blood pressure recovery. *International Journal of Psychophysiology* 2021;167:94-101.
158. Roddick CM, Chen FS. Effects of chronic and state loneliness on heart rate variability in women. *Annals of Behavioral Medicine* 2021;55(5):460-75.
159. Xu Z, Zhang D, Xu D. et al. Loneliness, depression, anxiety, and post-traumatic stress disorder among Chinese adults during COVID-19: a cross-sectional online survey. *Plos One* 2021;16(10):e0259012.
160. Sams N, Fisher DM, Mata-Greve F. et al. Understanding psychological distress and protective factors amongst older adults during the COVID-19 pandemic. *The American Journal of Geriatric Psychiatry* 2021;29(9):881-94.
161. Takács J, Katona ZB, Ihász F. A large sample cross-sectional study on mental health challenges among adolescents and young adults during the COVID-19 pandemic at-risk group for loneliness and hopelessness during the COVID-19 pandemic. *Journal of Affective Disorders* 2023;325:770-7.
162. Straus E, Norman SB, Tripp JC. Et al. Behavioral epidemic of loneliness in older US military veterans: results from the 2019-2020 national health and resilience in veterans study. *The American Journal of Geriatric Psychiatry* 2022;30(3):297-310.
163. Wang Y, Cui C, Zhang Y, Wang L. Factors associated with sleep quality among “left-behind women” in rural China: a cross-sectional study. Sleep and Breathing 2021;25:1603-11.
